# A metabolic perspective on polyploid invasion and the emergence of life histories: insights from a mechanistic model

**DOI:** 10.1002/ajb2.16387

**Published:** 2024-08-07

**Authors:** Silvija Milosavljevic, Felipe Kauai, Frederik Mortier, Yves Van de Peer, Dries Bonte

**Affiliations:** 1 Department of Plant Biotechnology and Bioinformatics, Ghent University, VIB - UGent Center for Plant Systems Biology, B-9052 Ghent, Belgium; 2 Department of Biology, Terrestrial Ecology Unit, Ghent University, Karel Lodewijk Ledeganckstraat 35, BE-9000 Ghent, Belgium; 3 Department of Biochemistry, Genetics and Microbiology, University of Pretoria, Pretoria, South Africa; 4 College of Horticulture, Nanjing Agricultural University, Nanjing, China

**Keywords:** Body Size, Energy Budgets, Individual-Based Model, Metabolism, Polyploidization

## Abstract

**Premise:**

Whole genome duplication (WGD, polyploidization) has been identified as a driver of genetic and phenotypic novelty, having pervasive consequences for the evolution of lineages. While polyploids are widespread, especially among plants, the long-term establishment of polyploids is exceedingly rare. Genome doubling results commonly in increased cell sizes and metabolic expenses which may be sufficient to modulate polyploid establishment in environments where their diploid ancestors thrive.

**Methods:**

We developed a mechanistic simulation model of photosynthetic individuals to test whether changes in size and metabolic efficiency allow autopolyploids to coexist with, or even invade, ancestral diploid populations. Central to the model is metabolic efficiency, which determines how energy obtained from size-dependent photosynthetic production is allocated to basal metabolism as opposed to somatic and reproductive growth. We expect neopolyploids to establish successfully if they have equal or higher metabolic efficiency as diploids, or by adapting their life history to offset metabolic inefficiency.

**Key results:**

Polyploid invasion was observed across a wide range of metabolic efficiency differences between polyploids and diploids. Establishment of polyploids in diploid populations occurred even when the former had a lower metabolic efficiency, which was facilitated by recurrent formation. Competition for nutrients is a major driver of population dynamics in this model. Perenniality did not qualitatively affect from which relative metabolic efficiency tetraploids tend to establish.

**Conclusions:**

Feedback between size-dependent metabolism and energy allocation generated size and age differences between ploidies. We demonstrate that even small changes in metabolic efficiency are sufficient for the establishment of polyploids.

## Introduction

Polyploids are organisms with multiple sets of chromosomes gained through whole genome duplication (WGD), typically following the fusion of unreduced gametes (Ramsey and Schemske 1998; Van de Peer et al. 2017). WGD is considered important for genome evolution and a common mode of plant speciation (Wood et al. 2009). Polyploidy is present in most domains of life, including animals (Otto and Whitton 2000; Mable et al. 2011) and fungi (Albertin and Marullo 2012; Todd et al. 2017), but it is particularly widespread across plants, where most of the current polyploid research is focused.

The apparent clustering of past WGD events around periods of environmental catastrophes, e.g., the Cretaceous-Paleogene boundary, suggests a relation between stress and polyploid emergence and establishment (Fawcett et al. 2009; Vanneste et al. 2014; Cannon et al. 2015; Lohaus and Van de Peer, 2016). Furthermore, polyploids often exhibit increased stress tolerance relative to their diploid counterparts (Chao et al. 2013; Yang et al. 2014; Rice et al. 2019; Marks et al. 2023) and undergo niche shifts and phenotypic differentiation, believed to contribute to successful establishment (Ramsey and Ramsey 2014; Kiedrzyński et al. 2021). Immediate phenotypic differentiation is often observed, such as in *Heuchera grossulariifolia*, where neotetraploids showed slower growth, and shorter flowering but larger flowers compared to diploids, contributing to niche shifts (Oswald and Nuismer 2011) and may relieve competitive pressures and aiding polyploid establishment. Despite generally being less fit than their diploid progenitors and potentially having cellular abnormalities and genomic instability (Clo et al. 2022), polyploids can establish through niche adaptations, selfing, and asexual reproduction (Fowler and Levin 1984; Rodriguez 1996; Novikova et al. 2022). Additionally, transitioning to perenniality extends the reproductive period window for polyploids, increasing their chances of successful mating (Gustafsson 1948; Van Drunen and Friedman 2022). Although many polyploids are perennial, whether perenniality is adaptive from a metabolic perspective and a consequence of high energetic demands in polyploids is unclear.

Metabolic rate increases allometrically, i.e., with body size making individual size a key biological feature influencing behavior and physiology (Kleiber 1932; Peters 1983; White et al. 2007; Isaac and Carbone 2010). These effects of body size cascade into effects on higher ecosystem functioning through its impact on life histories and the resulting population dynamics (Yvon-Durocher and Allen 2012; Price et al. 2010). Likely due to the extra DNA and chromosomes, the most common consequence of WGD is larger cell sizes (Ramsey and Schemske 2002; Comai 2005; Bomblies 2020), which is expected to increase the metabolic costs (Van Drunen and Husband 2019). These changes impact energy use and metabolism, and may particularly affect photosynthesis and gas exchange in plants (Bomblies 2020). Thus, longer cell cycles, and reduced metabolic and growth rates (Bennett 1972; Beaulieu et al. 2007), might lead to a lower overall metabolic efficiency in polyploids. Understanding how polyploids can invade a diploid population under these conditions can therefore provide important insights into the dynamics of mixed-ploidy populations, and contribute to our general understanding of ecosystem functioning more broadly.

The impact of metabolic processes on life history will depend on how acquired energy is allocated among different life functions, such as maintenance, growth, and reproduction. This allocation of energy is central to the Dynamic Energy Budget (DEB) theory (Kooijman 2009). In DEB models, the proportion of assimilated energy allocated to the soma includes basal metabolism and somatic growth, while the remaining energy supports maturity maintenance, development, and reproduction (Kooijman 2009; Martin et al., 2012). An individual’s metabolic efficiency is directly proportional to the differential distribution of energy to fulfill basic metabolic functions. Higher metabolic efficiency of an individual then means that less energy is required for basal metabolism, allowing more energetic investment in reproduction and contributing to fitness. Ecophysiological and DEB models have advanced understanding of species range forecasting and ecotoxicology (Jager et al. 2014; Monaco and McQuaid 2018; Strubbe et al. 2023). We believe that they also provide a solid framework to investigate how the expected changes in metabolic efficiency in polyploids affect their success.

Here, we use individual-based simulations to explore how changes in metabolic efficiency in emerging autopolyploids affect whether polyploids can establish a viable population while in competition with diploids. Body size, energy acquisition, and different metabolic efficiencies eventually shape individual life histories, population dynamics, and the potential for species or cytotypes to invade populations of others. We hypothesize that neopolyploids need a comparable or higher metabolic efficiency than diploids to successfully invade (and thus outcompete others) or establish (co-occur with) their diploid ancestors. Alternatively, polyploids might counterbalance lower metabolic efficiency through life history adaptations such as perenniality and delayed maturation (Van Drunen and Husband 2019). We explicitly consider feedback with the environment by including nutrients, where competition for those limited nutrients emerges as the growth of both cytotypes depends on the nutrient uptake and photosynthesis.

Furthermore, we model a scenario where WGD coincides with a transition from annual to perennial life histories to understand their adaptive value under different metabolic efficiencies, where an extended reproductive period might also increase competition for nutrients. By exploring different metabolic efficiency scenarios, the model aims to capture potential variations in the metabolic strategies of polyploids and their implications for population dynamics and polyploid establishment. To maintain focus on the elementary processes of energy allocation we restrict our approach to non-sexually reproducing organisms and explicitly test how energy allocation processes underlie life history changes (reproduction, aging, and death), including the commonly observed dominance of perennial life histories in polyploids (Mortier et al. 2024).

## Materials and Methods

**Figure 1:**
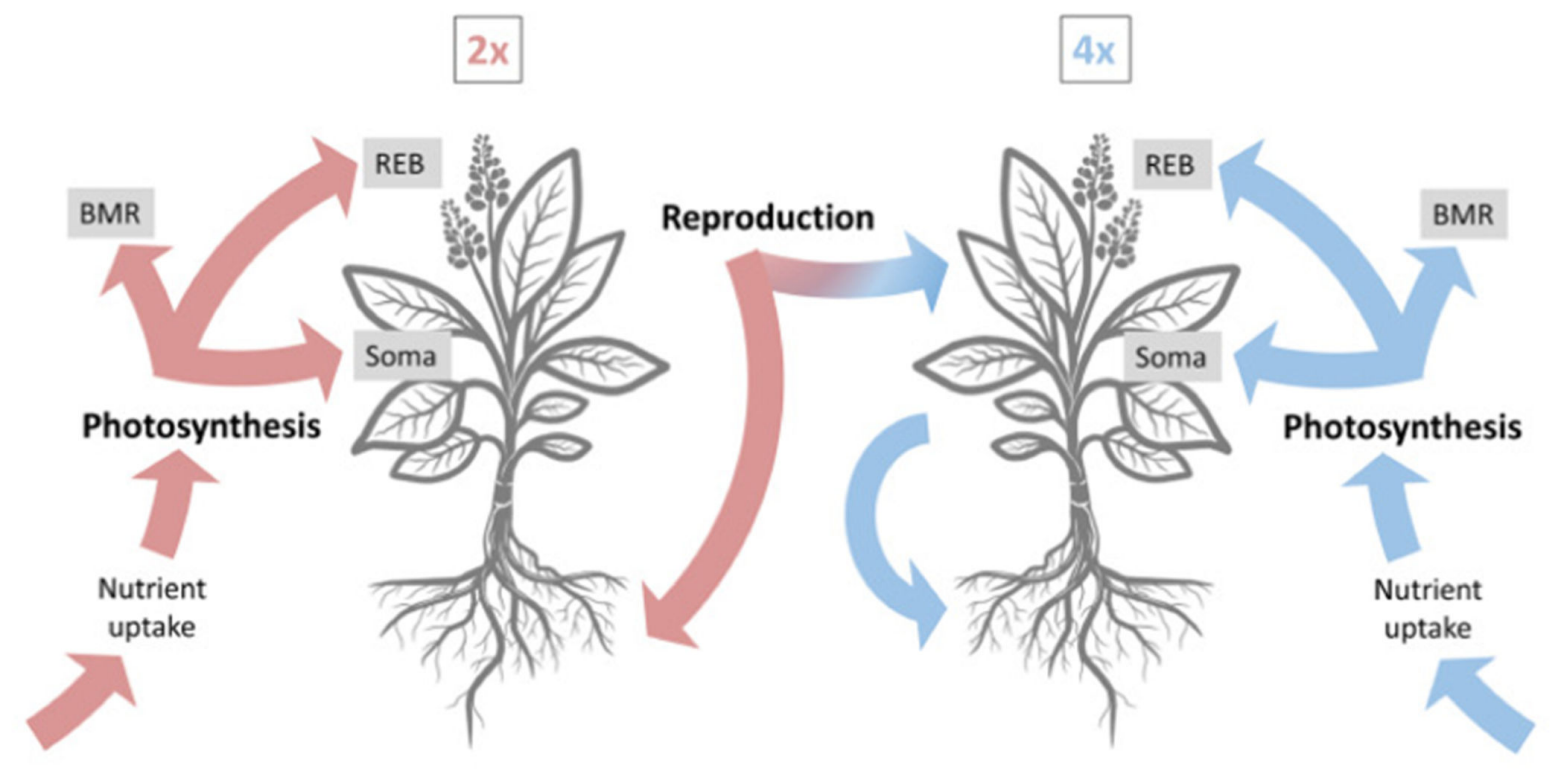
Schematic representation of the processes implemented in the model. All processes occur at a daily basis, except reproduction, which happens after 100 days (one season). Red colors are for diploids, and blue for tetraploids, throughout the manuscript. Notice that tetraploid individuals only produce tetraploid offspring. BMR – basal metabolic rate, Soma – somatic or vegetative growth, REB – reproductive energy budget.

We developed a consumer-nutrients, individual-based, and spatially explicit model to investigate the conditions under which polyploids can establish a viable population, leading to coexistence or invasion of the initial diploid population. The model is conceptually tailored to a diploid plant population, where autotetraploids emerge during reproduction. The population inhabits a square lattice of size *L* = 40, where each cell contains a certain number of resources that are replenished daily and consumed by the individuals for metabolic maintenance. The growth of individuals is proportional to the photosynthetic rate modulated by body mass and nutrient availability. The energy from photosynthesis is allocated to Basal Metabolic Rate (BMR) on one hand, and Somatic Growth (SOMA) and Reproductive Energy Budget (REB) on the other hand, in a fixed ratio which we denote *metabolic efficiency*. Energy towards somatic growth increases with body size, which feeds back to photosynthesis. The reproductive outcome of an individual is a function of its REB and takes place every 100-day interval, which we consider a *season*. Offspring from diploids can be either diploid or tetraploid, whereas tetraploids only produce tetraploid seeds. A schematic overview of the model is depicted in Fig. 1. We will briefly explain the model here but provide a more detailed description in Appendix S1 (see the Supplementary Data with this article), according to the ODD protocol (Overview, Design concepts, Details) for describing individual-based models (Grimm et al. 2006, 2010, 2020).

### Nutrient consumption and photosynthesis

Resources (nutrients) are replenished linearly daily with rate *r* (g/day), up to a maximum per cell unit *Rmax* (g/cell). Nutrient availability is thus auto-correlated in time. We model a single pool of resources, an abstraction of the limiting nutrients needed for plant growth, like nitrogen or phosphorus. The model is a discrete-time model where one time step corresponds to one day in the lifetime of a consumer. Individuals consume nutrients daily based on their photosynthetic rate, calculated using the relationship between plant mass *M (g)* and photosynthetic rate *Q (g/day)* from the Michaelis-Menten model studied by Hu et al. (2021):

(Eq. 1 Q=(QM×M)/(kQM+ϑ×M)

Where *QM = 32.8, kQM = 1.06*, and *ϑ = 0.94* are parameters derived for non-woody plants in Hu et al. (2021), reflecting the joint effect of mass and limiting nutrients on photosynthesis.

In compliance with metabolic theory, the photosynthetic rate, as the main metabolic rate considered here, is not dependent on the nutrient availability (Price et al. 2010). However, effective photosynthetic rate is limited by nutrient availability. Individuals compete for limited nutrients within each cell. Individuals experience contest competition for nutrients. If available nutrients are insufficient for an individual’s photosynthetic demands, the individual consumes all of the remaining nutrients in the cell.

### Metabolism and metabolic efficiency

Metabolic efficiency is defined as the ratio of energy allocated to BMR, SOMA, and REB. Energy and nutrient usage in diploids were taken to be 36:54:10 (BMR:SOMA:REB). This ratio was chosen based on the input from DEB theory parameters. In many species, the proportion of assimilated energy allocated to the soma is around 80-90%, while the proportion allocated to reproduction is then 10-20% (Kooijman 2009; Martin et al., 2012). We chose allocation to the reproductive budget to be 10%, and allocation to somatic growth to be 54%. These parameters reflect the proportion of dark respiration metabolism in plants compared to the total photosynthetic metabolism. Dark respiration reaches up to 50% of the total metabolism in highly respiring plants, for example, carnivorous plants (Hájek and Adamec, 2010), while respiration rates are found to be around 20-25% of the photosynthesis rates (McCree and Troughton, 1966) leading to the average value of 36% we chose. For convenience, we take this energy allocation distribution to be the reference metabolic efficiency from which deviations are measured.

To investigate the effects of different metabolic efficiencies on population dynamics and tetraploid establishment, the model explores scenarios where the metabolic efficiency of tetraploids is lower, equal, or higher than that of diploids. We assume differentiation in the metabolic efficiency as a trait that occurs immediately after WGD. Lower metabolic efficiency is modelled by increasing the proportion of energy allocated to BMR, at the expense of reproductive energy budget growth. This is achieved by altering the 36:54:10 ratio to reflect lower metabolic efficiencies in tetraploids—for instance, 37:54:9, indicating a 10% reduction in efficiency compared to diploids. Conversely, higher metabolic efficiency for polyploids is modelled by decreasing the allocation to BMR. This implies that polyploids allocate more to reproduction compared to diploids, resulting in higher polyploid fitness and invasion. As that is an expected and trivial result, we do not show the results of those simulations.

Following nutrient consumption (Equation1), energy is first allocated to BMR, then SOMA and REB with daily demands of *0.36Q, 0.54Q*, and *0.1Q*, respectively, for a diploid individual. Naturally, nutrient availability may prevent individuals from fulfilling these metabolic demands. Thus, if an individual does not acquire enough energy for basal metabolism during a day, it experiences mass loss, which results in premature death when the mass reaches zero. If, on the other hand, energy is not enough to fulfill REB’s requirements, nothing happens. However, this will lead to a decrease in the rate of accumulation of REB throughout the organism’s development, which may hinder its ability to successfully reproduce by the end of a growing season.

### Reproduction

Energy for reproduction is accumulated into the REB over all 100 days of the growing season. At the end of this period, all individuals produce seeds by subtracting energy units from their REB until it is depleted. The cost of a seed is equal to the seed size and each seed is initialized with a size sampled from a normal distribution with a mean of 0.5 g and a standard deviation of 0.05 g (chosen based on data presented in Moles et al. 2005 and Seed Information Database (SID), 2023). Seeds are initialized with a small probability of being polyploid when produced by diploids. For simplicity, we assume tetraploids can only produce tetraploid seeds, and seed sizes follow the same distribution as diploid seeds. Seed dispersal is modelled as random to the adjacent to its parent (nearest-neighbor dispersal) with no cost of dispersal. Also, as we base this model on plants, there is only movement due to seed dispersal. Finally, the modeled species is semelparous, so individuals die after reproduction.

### Polyploid formation

Seeds from diploid parents are modelled to be polyploid with a unique probability that is determined by a Beta distribution with shape parameters *α* = 2 and *β* = 40 (resulting in expected mean = 0.0476). Our chosen Beta distribution captures the zero-inflated nature of polyploid formation rates (Bretagnolle and Thompson 1995; Ramsey 2007) and the higher mean observed in asexual species by Kreiner and colleagues (2017b). This probability is applied to each reproducing diploid, for the whole duration of the simulation, unless specified differently. Hence, polyploid formation is a recurrent process at each reproduction event. Only under conditions of positive fitness populations of polyploids should grow from these low baseline population sizes.

#### Life history strategies

In addition to testing the impact of metabolic efficiency changes in polyploids, we also investigate the impact of different life history strategies. While perenniality has been demonstrated to be beneficial in polyploids by its impact on sexual reproduction (Van Drunen and Husband 2019), we here test its adaptive value in relation to differences in metabolic efficiencies and competition in polyploids versus diploids. We expand simulations with 1) all annuals to also simulate 2) all perennials, and 3) annual diploid and perennial polyploids. This last scenario is applicable if WGD causes a direct shift to perenniality. We model semelparous reproduction. Annuals in our model die at the end of the growing season regardless of reproductive success, whereas perennials can survive and reproduce in the subsequent seasons if they did not reproduce yet.

#### Computer simulations and data analysis

Simulations start with seeds introduced at an average density of one individual per cell (a total of 1600 seeds). Seed size is sampled from a normal distribution with a mean of 0.5 g and a standard deviation of 0.05 g. Simulations run for 10000 days (100 seasons). The parameter space tested includes reductions in the metabolic efficiency of tetraploids in comparison to that of the diploids with increments of 1% from 0 to 10% (from no difference between polyploids and diploids, to polyploids having 1, 2, …, 10% less metabolic efficiency than the diploids), for each life history strategy scenario. For exact initialization and parametrization of the model and tested scenarios, see the Appendix S1. During each simulation, we tracked changes in the number of individuals (total, diploids, and polyploids), nutrient levels per cell, individual sizes, and their REB. To track the self-sufficiency of the polyploid population, we also tracked whether the polyploid individuals are from diploid parents or from previously formed polyploids successfully reproducing. Under fitness advantages, polyploids will always increase from low numbers at the expense of their diploid ancestors, hence increasing in frequency. We thus use this invasion criterion to understand the emergence of adaptive strategies from changes in metabolic compartmentalization. Only under equalizing fitness mechanisms, coexistence is reached. The results presented are based on 10 independent runs for each parameter set. Sensitivity analyses tested the robustness of the results to changes in other model parameters. An overview of all parameters used is available in Table S1 of Appendix S1.

## Results

### Polyploid invasibility and population dynamics

**Figure 2:**
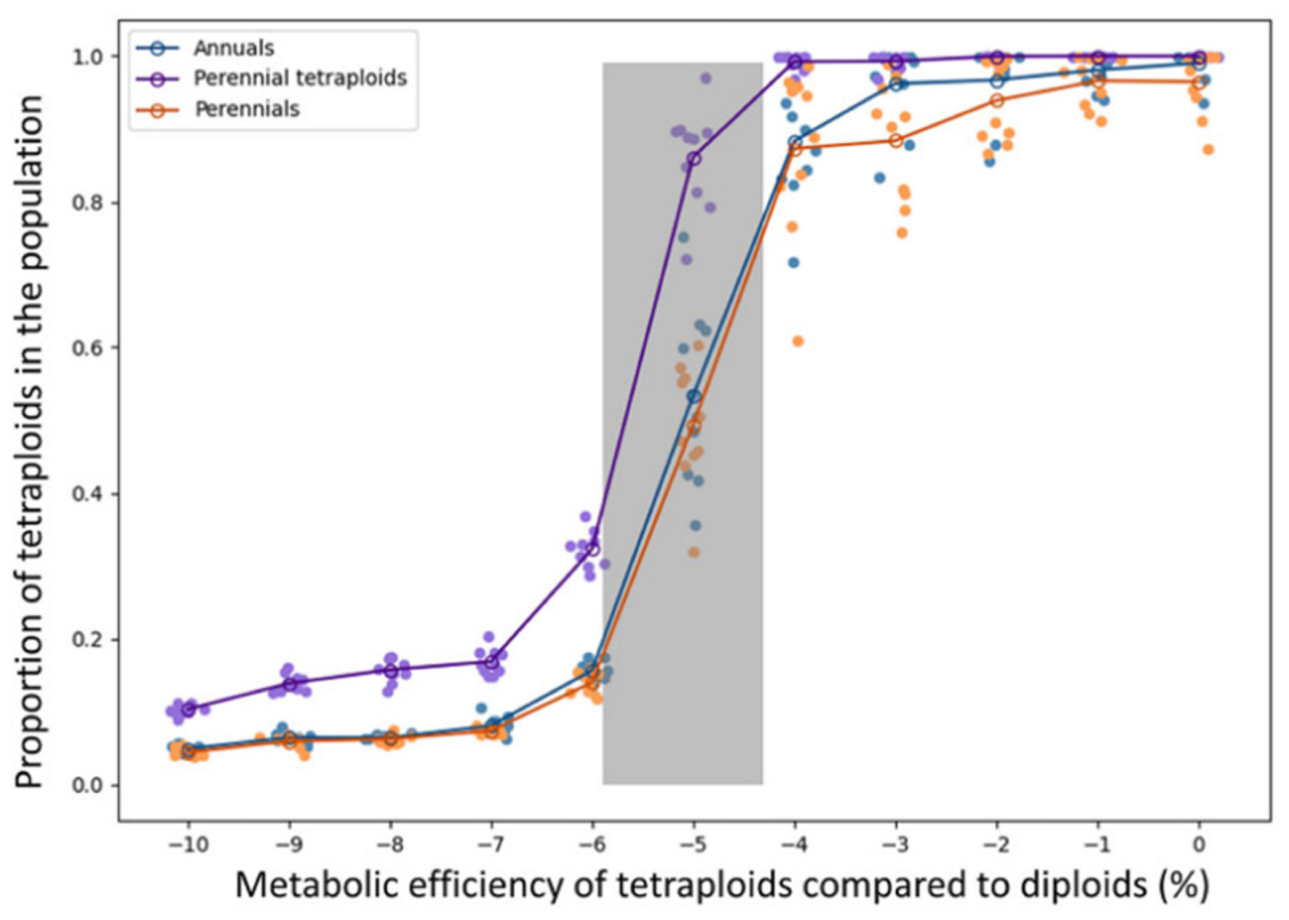
Proportion of tetraploid individuals across metabolic efficiencies of tetraploids in comparison to diploids. Results are shown for all annuals in blue, all perennials in orange, and tetraploid perennials in purple. Filled dots plot the mean polyploid proportion after 100 growing seasons for independent simulations with applied jitter along the x-axis to distinguish between the same metabolic efficiencies, with the open dots the mean for that metabolic efficiency and scenario. The grey-shaded area represents the zone where coexistence is observed.

We examined the final proportion of tetraploid individuals within the population across a range of metabolic efficiencies for all combinations of life history traits considered, i.e., only annuals, only perennials, and annual diploids with perennial tetraploids (Fig. 2). Simulations revealed that tetraploids quickly overtake the population when their metabolic efficiencies were at most 5% reduction, relative to the metabolic efficiency of the diploids (right side, Fig. 2). Analyses of the scenarios of all-annuals and all-perennials showed negligible differences. However, we found that when tetraploids are perennials in a population of annual diploids (purple in Fig. 2), invasion of tetraploids became feasible at even lower metabolic efficiencies, as indicated by the consistently high tetraploid proportions in the population by the end of simulations (100 seasons). Nonetheless, with reductions in metabolic efficiency greater than 5%, tetraploid invasion was not verified in any of the scenarios. In addition, the parameter space that leads to a temporal polyploid-diploid coexistence that is still apparent after 100 growing seasons is narrow (grey zone in Fig. 2).

**Figure 3:**
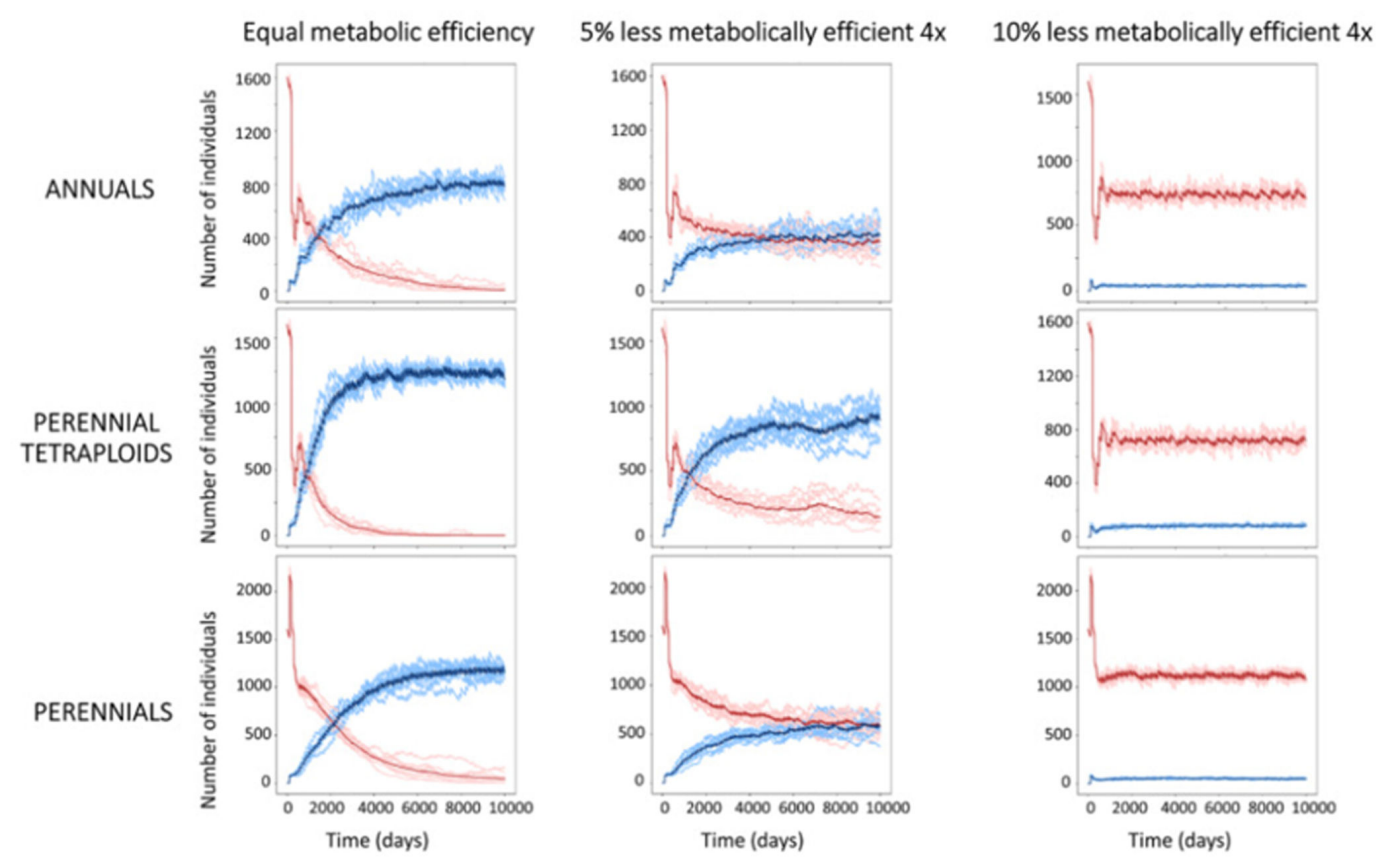
Population sizes across simulation time. Number of diploid (red) and tetraploid (blue) individuals through 10000 days, across simulated scenarios and metabolic efficiencies in the three life history scenarios. We plot metabolic efficiencies that are equal, tetraploids are 5% less efficient and 10% less efficient than diploids, respectively. The mean is plotted in a darker shade than all independent simulations.

Population dynamics over time (Fig. 3) revealed that polyploids with 10% lower metabolic efficiency than diploids failed to establish. The resulting population sizes are similar to those without polyploid formation (Appendix S2, Figs. S2, S3). Insights into which metabolic efficiencies enabled establishment were unaffected by the chosen Beta distribution, i.e. the polyploid formation rate (Appendix S2, Figs. S4, S5). Halving the polyploid formation rate from 4.76 to 2.38% broadened the zone of coexistence and shifted slightly to higher metabolic efficiencies (Appendix S2, Fig. S5b). The pivotal role of recurrent polyploid formation for enabling the polyploid dominance is substantiated by exploring scenarios with a single or a burst of ten growing seasons featuring polyploid formation, in contrast to the constant polyploid formation. This revealed that a single-season polyploid formation constrained establishment, and even with ten seasons of polyploid formation, successful establishment remained challenging unless polyploids had high metabolic efficiency in comparison to diploids (Appendix S2, Fig.S6).

These results hold for both the annual, perennial polyploids and perennial scenario despite changes in overall carrying capacities. Carrying capacity (equilibrium population size) increased by 50% for perennials (from ~800 to 1200; Fig. 3, compare the top and bottom rows). Contrary to our expectations, perenniality, and the resulting accumulation of resources and energy reserves over time, did not show to be adaptive in polyploids. In fact, as elucidated in Fig. 3 (bottom left), perenniality often postpones the invasion of tetraploids, due to an increased competition for nutrients among a larger pool of individuals.

### Size and age at maturity

**Figure 4:**
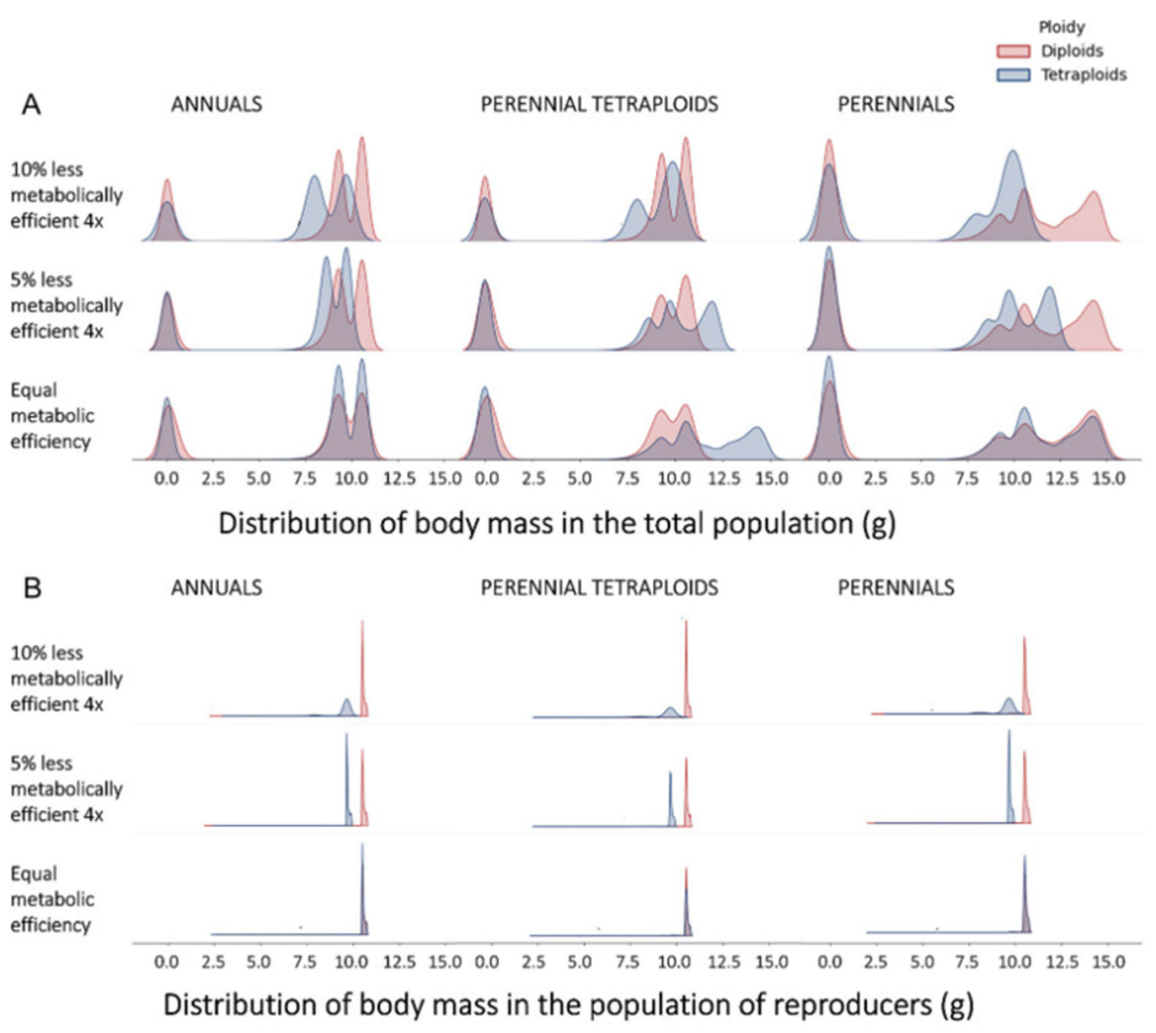
Distribution of individual mass across metabolic efficiencies and life history scenarios. A) Shows the masses of all individuals, whereas B) shows only the masses of individuals that reached maturity and successfully reproduced. Different rows correspond to the tested metabolic inefficiencies of tetraploids in comparison to diploids, with the top and middle rows where tetraploids are 10% and 5% less efficient than diploids, respectively, and the bottom row where they have an equal metabolic efficiency. Different columns correspond to the tested life history scenarios, with the first column corresponding to the annuals scenario, the second to the scenario where only tetraploids exhibit perenniality, and the third to the perennials scenario. Notice that red colors represent diploids and blue represent tetraploids.

We investigate the life history strategies that emerge in our simulations through the population’s distributions of size and age. Resource availability was crucial in determining the maximal biomass growth. Mass distributions exhibited a multimodal distribution (Fig. 4A), reflecting the distinct phases in individual development. That is, young individuals, such as seeds and seedlings, exhibit low masses, whereas individuals close to reproductive maturity have greater mass. As the simulation progresses, competitively superior individuals thrive by outcompeting others for nutrients, attaining substantially higher masses. Under the scenarios of all-annuals and all-perennials, mass distributions of both ploidies align closely when metabolic efficiency is similar between them (Fig. 4A, bottom row). However, for perennial polyploids and annual diploids, we verified asynchrony in the mass distributions, highlighting the greater accumulated somatic growth attained by tetraploids under perennial life histories. Additionally, under all scenarios, as the metabolic efficiency of tetraploids decreased, the masses of both reproducing and non-reproducing tetraploid individuals reduced significantly compared to diploids.

Noteworthy variations emerged between the annual and perennial life history scenarios. When all individuals were perennial (third column of Fig. 4A), an additional prominent peak emerged with individuals of high masses (above 12g). This peak was also present when only tetraploids are allowed to adopt perenniality (second column of Fig. 4), except when tetraploids were 10% less metabolically efficient than diploids. These distinctive high-mass peaks were absent among reproducing individuals (Fig. 4B), demonstrating the persistent presence of high-mass but non-reproducing individuals in the population. A reduction of 10% metabolic efficiency combined with perennial lifestyles showed the most detrimental for polyploid fitness and establishment.

**Figure 5:**
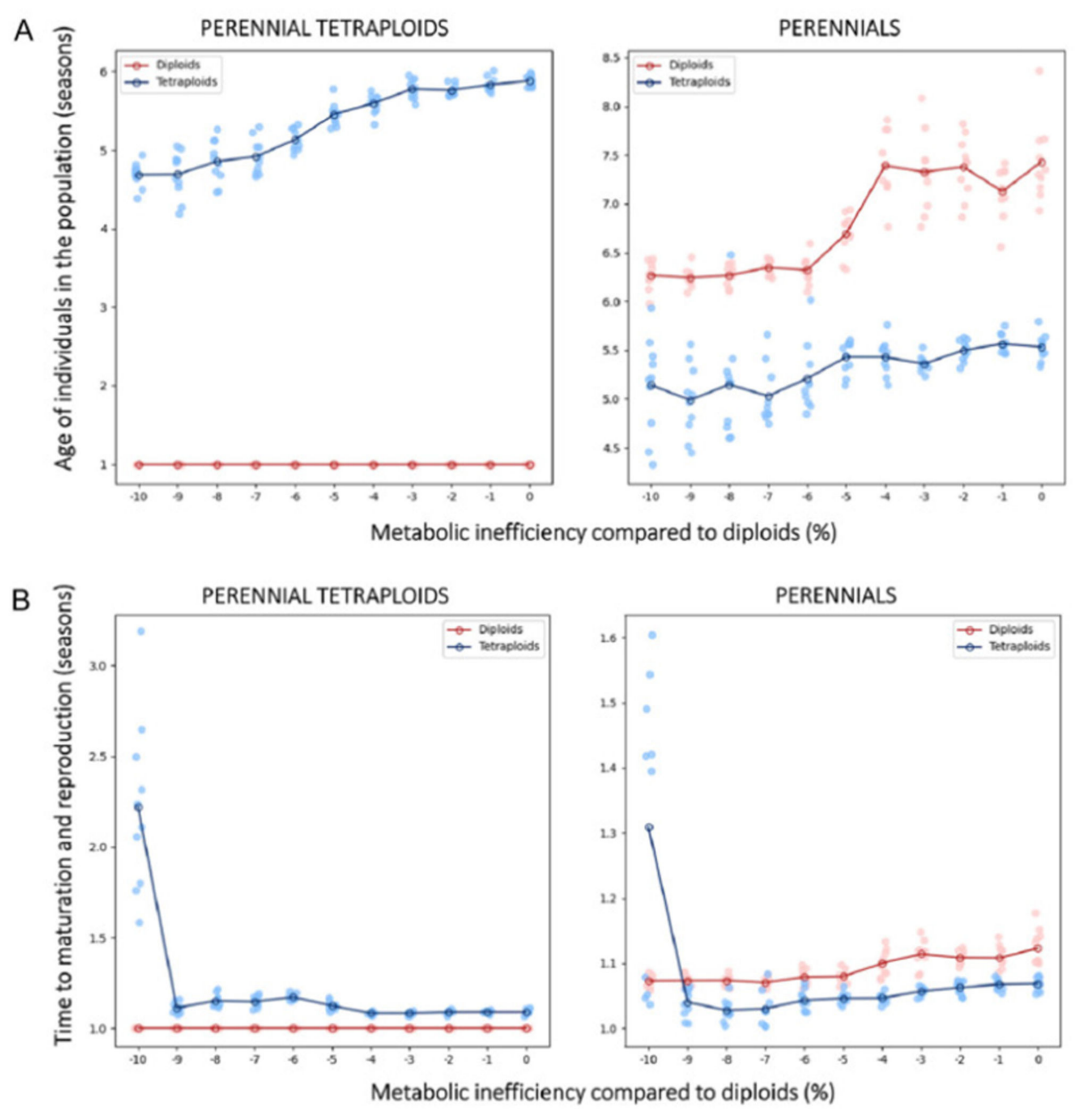
Ages of diploid and tetraploid individuals across simulated metabolic inefficiencies of tetraploids and life history strategies. Zero % metabolic inefficiency means the efficiency of tetraploids is equal to the efficiency of diploids, which is held constant in all simulations. A) Shows the average ages of all individuals, whereas B) shows the average ages of only the individuals which matured and successfully reproduced. The color of lines is differentiating between diploids (depicted in red) and tetraploids (depicted in blue). Different plots correspond to the life history scenarios, with the left one corresponding to the scenario where only tetraploids exhibit perenniality and the right one to the scenario where all individuals are perennials. Notice that these plots illustrate the mean of multiple simulations in darker color shades of red and blue with applied jitter along the x-axis for visibility, while the lighter shades are the independent simulations, and that “all annuals” scenario is not shown as there all the ages are one season.

In our simulations, perennials can persist over multiple years until successful reproduction or death by starvation, while annuals age within one growing season. We therefore explored the age structure of the population across the simulated parameter set and found that the average age of individuals typically exceeds four seasons (Fig. 5A). While the figure presents average ages, the simulated populations do encompass significantly older individuals (Appendix S2, Fig. S7). Very low metabolic efficiency in tetraploids extended the average time to reproduction (Fig. 5B), but across all metabolic efficiencies, most individuals reproduced after one growing season. Therefore, a large portion of the population consisted of old individuals that died off without reproducing. Similarly, in scenarios where only tetraploids adopt perenniality (even in the presence of tetraploid metabolic inefficiencies; see higher), age to reproduction is generally low, averaging close to one year. In line with the analysis of size dynamics (Fig. 4, successful reproducers are not the largest in the population), we showed that individuals that persist for multiple seasons grow larger/taller but do not achieve successful reproduction. We found that successful individuals in our model have emerging annuality, indicating perenniality to be merely maladaptive.

## Discussion

To better understand the eco-evolutionary dynamics of mixed ploidy populations, we developed an individual-based modeling grounded in DEB theory. We deliberately focused on feedback from size and metabolic efficiency to life history because of its well-known impact on population dynamics (Price et al. 2010; Kooijman 2009). We show that polyploid establishment and invasion are possible when their metabolic efficiency is lower than that of diploids but highly dependent on the recurrence of polyploid formation. We also demonstrated that perenniality does not inherently confer advantages for asexual neopolyploids, but that it greatly changes the population structure and imposes increased intra- and intercytotype competition for nutrients. These insights provide a fresh look at the mechanisms underlying polyploid success and complement other theoretical approaches with a usual strong focus on minority cytotype exclusion and other eco-evolutionary principles (e.g., Levin 1975; Fowler and Levin 1984; Felber 1991; Van Dijk and Bijlsma 1994; Rodriguez 1996; Suda and Herben 2013; Fowler and Levin 2016; Griswold 2021; Kauai et al. 2023).

Invasion theory teaches us that neopolyploids must demonstrate higher fitness than their ancestor diploid population at low densities. Coexistence between these cytotypes is only possible when fitness becomes equalized, or when life history trade-offs become persistent (Mortier et al. 2024). We show that neopolyploids with metabolic efficiencies larger, equal, or only slightly less than their diploid ancestors can successfully establish and invade the population. A reduction in metabolic efficiency of more than 5% in polyploids relative to diploids hampers polyploid establishment in competition with their ancestor. This emphasizes the need for real plant polyploids to evolve adaptations that either counterbalance the costs associated with larger size and reduced metabolism to equalize inherent fitness differences with their diploid ancestors or reduce competition with their ancestor by evolving niche differentiation to enable coexistence (Mortier et al. 2024).

A reduction in metabolic efficiency in polyploids can result from a multitude of cellular processes triggered by genome doubling, including cell size increase, genomic instability, and changes in gene expression and epigenetics (Comai 2005). In plants, CO_2_ exchange rates, a measure for basal metabolic processes, tend to decrease with increasing ploidal levels. For example, tetraploids average about 11% lower CO_2_ exchange rates than diploids (Levin 1983), indicating reduced efficiency. This aligns with our model assumptions and results. Studies on artificial colchicine-induced autopolyploids of *Arabidopsis thaliana* revealed differences in metabolites related to the tricarboxylic acid (TCA) cycle and the γ-aminobutyric acid (GABA), which could have adaptive consequences for the polyploids due to the diverse functional roles of these metabolites (Vergara et al. 2016). Such a decrease in metabolites of the primary metabolism suggests shifts in metabolic efficiency, providing an opportunity for neotetraploids with minor physiological differences to diverge from the ancestral diploids in ecological interactions, as concluded also by Vergara et al. 2016.

Conversely, polyploids with higher metabolic efficiencies outcompete their diploid ancestors. For example, *Beta vulgaris* (Levin 1983) and *Festuca arundinacea* (Byrne et al. 1981) exhibit higher CO_2_ fixation rates. Also, *Jasione maritima* var. *maritima* neotetraploids show higher chlorophyll fluorescence parameters, an overall indicator of photosynthesis, than diploids (Siopa et al. 2020). However, this increased efficiency is not reflected in increased biomass production, likely due to allocating to defense mechanisms and starch accumulation instead of growth (Castro et al. 2023). The lack of gigas effect in this case aligns with the results of our model, where polyploids are smaller due to lower metabolic efficiency and increased competition for nutrients (see below).

Recurrent polyploid formation emerges as a significant driver of establishment success, as shown in previous models (for example Felber 1991; Fowler and Levin 2016). Our results also suggest that episodic bursts of polyploid formation are not permissive for successful establishment, unless neopolyploids have high metabolic efficiencies. Recurrent polyploidy, reflected in this model by allowing polyploid offspring in each reproductive trial, is found in most studied plant systems (Soltis and Soltis 1999; Shimizu-Inatsugi et al. 2009; Kolář et al. 2012; Čertner et al. 2017). However, clear empirical data on the variation of unreduced gamete formation are lacking, which is why we choose a Beta probability distribution with mean of about 4.76% to reflect insights from Ramsey (2007) and Kreiner et al. (2017a).

Diploid and autotetraploid plants have different growth rates and life histories (Levin 1983). Perenniality is common in polyploid taxa (Müntzing 1936; Stebbins 1938; Gustafsson 1948; Rice et al. 2019). For example, an autotetraploid strain of *Zea mays* is perennial, while diploid maize is annual, a pattern also seen in *Eragrostis* and *Nasturtium* (Müntzing 1936). In *Oryza punctata* tetraploids survive 2-9 years, while diploids rarely live beyond one year (Sano 1980). Such greater longevity in tetraploids is also observed in *Trifolium* species (Frame et al. 1976) and *Medicago* (Small 2011), although the correlation between perenniality and polyploidy is less clear in angiosperms overall (Van Drunen and Husband 2019). Due to associations of perenniality with polyploidy and its theoretical benefits for reproductive assurance, we investigated scenarios with annuality vs. perenniality in our model. Contrary to previous assumptions (Rice et al. 2019; Van Drunen and Friedman 2022), our study challenges the notion that perenniality inherently confers reproductive advantages, solely based on metabolic principles applied here. When both diploid and tetraploid individuals are allowed to be perennial, tetraploid invasion is feasible across the same parameter space as if the individuals were annual. However, perenniality often delays the invasion of tetraploids due to intensified competition for resources, as the prolonged presence of individuals attempting reproduction over multiple seasons reduces the number of generations within the simulated 100 growing seasons.

Our model shows that perennial polyploids which emerged in annual diploids population can establish with lower metabolic efficiencies than when having the same life history strategy as the diploids. The exploration of size and age at maturity provides insight into these results. We observed multimodal mass distributions that highlight distinct life-history groups, where seeds and young individuals have low body masses and older individuals that accumulated more nutrients have grown larger. High competition leads to lower masses in metabolically inefficient polyploids, resulting in smaller sizes compared to diploids. Polyploid size at maturation and reproduction is eventually lower than the size of diploids as well. The age and size structure further reveals the importance of metabolic efficiency and confirms that perenniality is not always beneficial, due to the competition for nutrients between higher numbers of individuals that emerge in the perennial scenario. Only fast growing, on average annual, individuals reproduce, while most individuals living for multiple seasons grow in size and compete for resources, but without contributing to reproduction (“living deads”). Thus, perennial individuals in our model then tend to grow to high masses but rarely reach maturity and reproductive capability. Essentially, the need to compensate for inefficiency propels successful individuals towards swift reproduction. This emphasizes the trade-offs and challenges faced by tetraploids with reduced metabolic efficiency in this model, hindering their subpopulation growth due to the inability to invest accumulated energy directly in reproduction.

In nature, organisms may overcome challenges of limited nutrients and competition by adapting their resource allocation strategy and investing immediately into reproduction. Due to distinct resource allocation into BMR, soma, and REB, our model results are contrary to the study showing that larger individuals are more fit than smaller ones (Malerba et al. 2018). Our findings also do not confirm the emergence of larger sizes of polyploids observed in nature (Bomblies 2020). It can be hypothesized that other processes during development or advantages of larger sizes that overrule the metabolic costs (Malerba et al. 2018) and lead to niche differences are important drivers of size differences between polyploids and their ancestors in real polyploid species, as opposed to the model presented here. Instead, successful reproduction in our model is driven by rapid growth and efficient resource use, with smaller individuals being competitively superior in nutrient-limited environments.

This model is limited to autopolyploids as the polyploidization happens within a population. It would be interesting to explore the effects of allopolyploidization in future work, with allopolyploids emerging from ancestors with different metabolic capacities. Depending on the assumptions made about the energy allocation and differentiation from the diploids, one might expect the same or diverging results. Although disentangling the effects of hybridization and genome duplication in allopolyploids is complex, phenotypic plasticity and the potential for transgressive phenotypes gained after allopolyploidization can be beneficial and therefore interesting to take into account (Qiu et al. 2020).

## Conclusions

In conclusion, our study showcases the complex interplay between allometric scaling of metabolism, metabolic efficiency, and life history strategies in autopolyploid establishment. The insights derived from our mechanistic modeling approach emphasize the importance of integrating ecophysiological insights to elucidate long-standing questions about polyploid establishment (Soltis et al., 2016). By integrating ecophysiological insights with population modeling, we explore a potential central role of metabolism influencing autopolyploid success. Our results point towards polyploid establishment and invasion being feasible under various conditions of metabolic efficiency and photosynthetic rates, even if metabolic efficiency decreases in polyploids, it should not induce a direct paradox to the polyploid persistence. In addition, model parameters such as photosynthetic rate and size are measurable in real systems allowing predictions from our study to be tested and validated in future research.

## Supplementary Material

Appendix 1

Appendix 2

## Data Availability

All relevant data are within the manuscript and its electronic supporting material (Appendix S1 and S2). The code developed for running the simulations is available on GitHub at https://github.com/silvijamilosavljevic/polyploidy-model-metabolic-perspective.

## References

[R1] Albertin W, Marullo P (2012). Polyploidy in fungi: evolution after whole-genome duplication. Proceedings of the Royal Society, B, Biological Sciences.

[R2] Beaulieu JM, Leitch IJ, Knight CA (2007). Genome Size Evolution in Relation to Leaf Strategy and Metabolic Rates Revisited. Annals of Botany.

[R3] Bennett MD (1972). Nuclear DNA content and minimum generation time in herbaceous plants. Proceedings of the Royal Society of London, B, Biological Sciences.

[R4] Bomblies K (2020). When everything changes at once: finding a new normal after genome duplication. Proceedings of the Royal Society, B, Biological Sciences.

[R5] Bretagnolle F, Thompson JD (1995). Gametes with the somatic chromosome number: mechanisms of their formation and role in the evolution of autopolyploid plants. New Phytologist.

[R6] Byrne MC, Nelson CJ, Randall DD (1981). Ploidy Effects on Anatomy and Gas Exchange of Tall Fescue Leaves. Plant Physiology.

[R7] Cannon SB, McKain MR, Harkess A, Nelson MN, Dash S, Deyholos MK, Peng Y (2015). Multiple Polyploidy Events in the Early Radiation of Nodulating and Nonnodulating Legumes. Molecular Biology and Evolution.

[R8] Castro M, Celeste Dias M, Loureiro J, Husband BC, Castro S (2023). Competitive ability, neopolyploid establishment and current distribution of a diploid–tetraploid plant complex. Oikos.

[R9] Chao D-Y, Dilkes B, Luo H, Douglas A, Yakubova E, Lahner B, Salt DE (2013). Polyploids Exhibit Higher Potassium Uptake and Salinity Tolerance in Arabidopsis. Science.

[R10] Clo J, Padilla-García N, Kolář F (2022). Polyploidization as an opportunistic mutation: The role of unreduced gametes formation and genetic drift in polyploid establishment. Journal of Evolutionary Biology.

[R11] Comai L (2005). The advantages and disadvantages of being polyploid. Nature Reviews Genetics.

[R12] Čertner M, Fenclová E, Kúr P, Kolář F, Koutecký P, Krahulcová A, Suda J (2017). Evolutionary dynamics of mixed-ploidy populations in an annual herb: dispersal, local persistence and recurrent origins of polyploids. Annals of Botany.

[R13] Fawcett JA, Maere S, Van de Peer Y (2009). Plants with double genomes might have had a better chance to survive the Cretaceous–Tertiary extinction event. Proceedings of the National Academy of Sciences, USA.

[R14] Felber F (1991). Establishment of a tetraploid cytotype in a diploid population: Effect of relative fitness of the cytotypes. Journal of Evolutionary Biology.

[R15] Fowler NL, Levin DA (1984). Ecological Constraints on the Establishment of a Novel Polyploid in Competition with Its Diploid Progenitor. The American Naturalist.

[R16] Fowler NL, Levin DA (2016). Critical factors in the establishment of allopolyploids. American Journal of Botany.

[R17] Frame J, Harkess RD, Hunt IV (1976). The effect of variety and fertilizer nitrogen level on red clover production. Grass and Forage Science.

[R18] Grimm V, Berger U, Bastiansen F, Eliassen S, Ginot V, Giske J, Goss-Custard J (2006). A standard protocol for describing individual-based and agent-based models. Ecological Modelling.

[R19] Grimm V, Berger U, DeAngelis DL, Polhill JG, Giske J, Railsback SF (2010). The ODD protocol: A review and first update. Ecological Modelling.

[R20] Grimm V, Railsback SF, Vincenot CE, Berger U, Gallagher C, DeAngelis DL, Edmonds B (2020). The ODD Protocol for Describing Agent-Based and Other Simulation Models: A Second Update to Improve Clarity, Replication, and Structural Realism. Journal of Artificial Societies and Social Simulation.

[R21] Griswold CK (2021). The effects of migration load, selfing, inbreeding depression, and the genetics of adaptation on autotetraploid versus diploid establishment in peripheral habitats. Evolution.

[R22] Gustafsson ÅKE (1948). Polyploidy, Life-Form and Vegetative Reproduction. Hereditas.

[R23] Hájek T, Adamec L (2010). Photosynthesis and dark respiration of leaves of terrestrial carnivorous plants. Biologia.

[R24] Hu H-J, Xu K, He L-C, Wang G-X (2021). A model for the relationship between plant biomass and photosynthetic rate based on nutrient effects. Ecosphere.

[R25] Isaac NJB, Carbone C (2010). Why are metabolic scaling exponents so controversial? Quantifying variance and testing hypotheses. Ecology Letters.

[R26] Jager T, Barsi A, Hamda NT, Martin BT, Zimmer EI, Ducrot V (2014). Dynamic energy budgets in population ecotoxicology: Applications and outlook. Ecological Modelling.

[R27] Kauai F, Mortier F, Milosavljevic S, Van de Peer Y, Bonte D (2023). Neutral processes underlying the macro eco-evolutionary dynamics of mixed-ploidy systems. Proceedings of the Royal Society, B, Biological Sciences.

[R28] Kiedrzyński M, Zielińska KM, Jedrzejczyk I, Kiedrzyńska E, Tomczyk PP, Rewicz A, Rewers M (2021). Tetraploids expanded beyond the mountain niche of their diploid ancestors in the mixed-ploidy grass Festuca amethystina L. Scientific Reports.

[R29] Kleiber M (1932). Body size and metabolism. Hilgardia.

[R30] Kolář F, Fér T, Štech M, Trávníček P, Dušková E, Schönswetter P, Suda J (2012). Bringing Together Evolution on Serpentine and Polyploidy: Spatiotemporal History of the Diploid-Tetraploid Complex of Knautia arvensis (Dipsacaceae). PLOS ONE.

[R31] Kooijman B (2009). Dynamic Energy Budget Theory for Metabolic Organisation.

[R32] Kreiner JM, Kron P, Husband BC (2017a). Evolutionary Dynamics of Unreduced Gametes. Trends in Genetics.

[R33] Kreiner JM, Kron P, Husband BC (2017b). Frequency and maintenance of unreduced gametes in natural plant populations: associations with reproductive mode, life history and genome size. New Phytologist.

[R34] Levin DA (1975). Minority Cytotype Exclusion in Local Plant Populations. TAXON.

[R35] Levin DA (1983). Polyploidy and Novelty in Flowering Plants. The American Naturalist.

[R36] Lohaus R, Van de Peer Y (2016). Of dups and dinos: evolution at the K/Pg boundary. Current Opinion in Plant Biology.

[R37] Mable BK, Alexandrou MA, Taylor MI (2011). Genome duplication in amphibians and fish: an extended synthesis. Journal of Zoology.

[R38] Malerba ME, Palacios MM, Marshall DJ (2018). Do larger individuals cope with resource fluctuations better? An artificial selection approach. Proceedings of the Royal Society, B, Biological Sciences.

[R39] Marks RA, Delgado P, Makonya GM, Cooper K, VanBuren R, Farrant JM (2023). Polyploidy enhances desiccation tolerance in the grass Microchloa caffra. BioRxiv.

[R40] Martin BT, Zimmer EI, Grimm V, Jager T (2012). Dynamic Energy Budget theory meets individual-based modelling: a generic and accessible implementation. Methods in Ecology and Evolution.

[R41] McCree KJ, Troughton JH (1966). Prediction of Growth Rate at Different Light Levels from Measured Photosynthesis and Respiration Rates. Plant Physiology.

[R42] Moles AT, Ackerly DD, Webb CO, Tweddle JC, Dickie JB, Pitman AJ, Westoby M (2005). Factors that shape seed mass evolution. Proceedings of the National Academy of Sciences, USA.

[R43] Monaco CJ, McQuaid CD (2018). Applicability of Dynamic Energy Budget (DEB) models across steep environmental gradients. Scientific Reports.

[R44] Mortier F, Bafort Q, Milosavljevic S, Kauai F, Prost Boxoen L, Van de Peer Y, Bonte D (2024). Understanding polyploid establishment: temporary persistence or stable coexistence?. Oikos.

[R45] Müntzing A (1936). The Evolutionary Significance of Autopolyploidy. Hereditas.

[R46] Novikova PY, Kolesnikova UK, Scott AD (2022). Ancestral self-compatibility facilitates the establishment of allopolyploids in Brassicaceae. Plant Reproduction.

[R47] Oswald BP, Nuismer SL (2011). Neopolyploidy and Diversification in *Heuchera grossulariifolia*. Evolution.

[R48] Otto SP, Whitton J (2000). Polyploid incidence and evolution. Annual Review of Genetics.

[R49] Peters RH (1983). Cambridge Studies in Ecology.

[R50] Price CA, Gilooly JF, Allen AP, Weitz JS, Niklas KJ (2010). The metabolic theory of ecology: prospects and challenges for plant biology. New Phytologist.

[R51] Qiu T, Liu Z, Liu B (2020). The effects of hybridization and genome doubling in plant evolution via allopolyploidy. Molecular Biology Reports.

[R52] Ramsey J (2007). Unreduced gametes and neopolyploids in natural populations of Achillea borealis (Asteraceae). Heredity.

[R53] Ramsey J, Ramsey TS (2014). Ecological studies of polyploidy in the 100 years following its discovery. Philosophical Transactions of the Royal Society, B, Biological Sciences.

[R54] Ramsey J, Schemske DW (2002). Neopolyploidy in Flowering Plants. Annual Review of Ecology and Systematics.

[R55] Ramsey J, Schemske DW (1998). Pathways, mechanisms, and rates of polyploid formation in flowering plants. Annual Review of Ecology and Systematics.

[R56] Rice A, Šmarda P, Novosolov M, Drori M, Glick L, Sabath N, Meiri S (2019). The global biogeography of polyploid plants. Nature Ecology and Evolution.

[R57] Rodriguez DJ (1996). A Model for the Establishment of Polyploidy in Plants. The American Naturalist.

[R58] Sano Y (1980). Adaptive strategies compared between the diploid and tetraploid forms of Oryza punctata. The Botanical Magazine Tokyo.

[R59] SER, INSR, RBGK (2023). Seed Information Database (SID).

[R60] Shimizu-Inatsugi RIE, Lihová J, Iwanaga H, Kudoh H, Marhold K, Savolainen O, Watanabe K (2009). The allopolyploid Arabidopsis kamchatica originated from multiple individuals of Arabidopsis lyrata and Arabidopsis halleri. Molecular Ecology.

[R61] Siopa C, Dias MC, Castro M, Loureiro J, Castro S (2020). Is selfing a reproductive assurance promoting polyploid establishment? Reduced fitness, leaky self-incompatibility and lower inbreeding depression in neotetraploids. American Journal of Botany.

[R62] Small E (2011). Alfalfa and relatives: evolution and classification of Medicago.

[R63] Soltis DE, Soltis PS (1999). Polyploidy: recurrent formation and genome evolution. Trends in Ecology and Evolution.

[R64] Soltis DE, Visger CJ, Marchant DB, Soltis PS (2016). Polyploidy: Pitfalls and paths to a paradigm. American Journal of Botany.

[R65] Stebbins GL (1938). Cytological Characteristics Associated with the Different Growth Habits in the Dicotyledons. American Journal of Botany.

[R66] Strubbe D, Jiménez L, Barbosa AM, Davis AJS, Lens L, Rahbek C (2023). Mechanistic models project bird invasions with accuracy. Nature Communications.

[R67] Suda J, Herben T (2013). Ploidy frequencies in plants with ploidy heterogeneity: fitting a general gametic model to empirical population data. Proceedings of the Royal Society, B, Biological Sciences.

[R68] Todd RT, Forche A, Selmecki A (2017). Ploidy Variation in Fungi: Polyploidy, Aneuploidy, and Genome Evolution. Microbiology Spectrum.

[R69] Van De Peer Y, Mizrachi E, Marchal K (2017). The evolutionary significance of polyploidy. Nature Reviews Genetics.

[R70] Van Dijk P, Bijlsma R (1994). Simulations of flowering time displacement between two cytotypes that form inviable hybrids. Heredity.

[R71] Van Drunen WE, Friedman J (2022). Autopolyploid establishment depends on life-history strategy and the mating outcomes of clonal architecture. Evolution.

[R72] Van Drunen WE, Husband BC (2019). Evolutionary associations between polyploidy, clonal reproduction, and perenniality in the angiosperms. New Phytologist.

[R73] Vanneste K, Maere S, Van de Peer Y (2014). Tangled up in two: a burst of genome duplications at the end of the Cretaceous and the consequences for plant evolution. Philosophical Transactions of the Royal Society, B, Biological Sciences.

[R74] Vergara F, Kikuchi J, Breuer C (2016). Artificial Autopolyploidization Modifies the Tricarboxylic Acid Cycle and GABA Shunt in Arabidopsis thaliana Col-0. Scientific Reports.

[R75] White CR, Cassey P, Blackburn TM (2007). Allometric Exponents Do Not Support A Universal Metabolic Allometry. Ecology.

[R76] Wood TE, Takebayashi N, Barker MS, Mayrose I, Greenspoon PB, Rieseberg LH (2009). The frequency of polyploid speciation in vascular plants. Proceedings of the National Academy of Sciences, USA.

[R77] Yang PM, Huang QC, Qin GY, Zhao SP, Zhou JG (2014). Different drought-stress responses in photosynthesis and reactive oxygen metabolism between autotetraploid and diploid rice. Photosynthetica.

[R78] Yvon-Durocher G, Allen AP (2012). Linking community size structure and ecosystem functioning using metabolic theory. Philosophical Transactions of the Royal Society, B, Biological Sciences.

