# Supplementary material for: A metabolic perspective on polyploid invasion and the emergence of life histories: insights from a mechanistic model
Source: Am J Bot. Author manuscript; Available in PMC 2025 Feb 1. (PMC7616395; doi:10.1002/ajb2.16387)
Supplement: Appendix 1 [file EMS198114-supplement-Appendix_1.docx]

# Appendix S1

This model description follows the ODD protocol (Overview, Design concepts, Details) for describing individual-based models (Grimm et al. 2006, 2010, 2020).

## ***Purpose —***

Our aim was to investigate how autopolyploid establishment in a diploid population is influenced by differences in metabolic efficiencies and life history strategies. We accomplished this by employing a mechanistic, individual-based model (IBM) grounded in consumer-resource dynamics. We determined the parameter combinations that facilitate polyploid establishment, coexistence, and invasion.

By applying an IBM, we successfully incorporated variations in polyploid offspring formation and stochasticity within our model. The individuals’ ploidy levels are categorized as diploid and tetraploid, without explicitly representing the underlying genome. This approach, coupled with the assumption of clonal reproduction, implies that our results can be interpreted both for asexually reproducing species and females of a sexual species that does not suffer from pollen limitation. Other interpretations are also viable, depending on the ploidy levels considered (e.g. haploids with emerging diploids).

## ***Entities, state variables, and scales —***

### *The consumers —*

The consumer species is individually modeled and has the following state variables:

-ploidy: set to have values 2 or 4 to imitate polyploidization to tetraploids within a diploid species

-*W0* (g): the seed mass of an individual

-*W* (g): referring to the current somatic mass of an individual

-*ER* (g): mass of the reproductive energy budget, which is how much of the body size can be invested in reproduction

-*CM* (g/day): consumption, the mass of the nutrients that should be consumed during a day, calculated for the individual’s total body mass (W+ER), based on Hu et al. (2021) photosynthetic rate prediction for non-woody plants

-nutrient allocation ratio: the total mass of the consumed nutrients within a day (CM) is used for basal metabolic maintenance (BM), then somatic growth (SOMA), and then reproductive energy budget growth (REB). Based on Hu et al. (2021), the allocation for diploid individuals is set to be 36 BM: 54 SOMA: 10 REB. For tetraploid individuals, this allocation ratio changes in order to simulate higher (lower BM and higher REB) or lower (higher BM and lower REB) metabolic efficiency.

-birthday and age (days): referring to the age, important for simulations of perennial life cycles as they might live for several seasons

-parent ploidy: important for tetraploid emergence estimation as tetraploids may have diploid or tetraploid parents

-*x* and *y* coordinate

### *The landscape —*

The landscape is a lattice of 40 × 40 cells, where each cell has a certain amount of resources (nutrients), represented as mass in grams. The edges of the landscape are not wrapped so there can be an effect on the seed dispersal. A cell is defined by the following state variables:

-*R0* (g): the initial amount of the nutrients. Each cell is initialized with 1 g of resources, as an abstraction of the main nutrients needed for plant growth like nitrogen and phosphorus.

-*r* (g/day): growth speed of the nutrients. Nutrients are growing linearly each day by 1 g (green shaded box in Fig. S1).

-*Rmax* (g): maximum amount of nutrients, 10 g in all cells.

Nutrient availability is then auto-correlated in time.

### *Scales —*

Seed dispersal is the sole form of movement considered in the model, drawing inspiration from sessile plant species. Given that the primary focus of this model was to examine a diploid population with emerging autopolyploids, the landscape size was intentionally kept relatively small. Seed dispersal was confined to the nearest neighbor cells. Each time step corresponds to one day, and a growth season of 100 days was used as the timeframe for reproduction opportunities. All simulations were conducted over 10000 time steps, equivalent to 100 seasons.


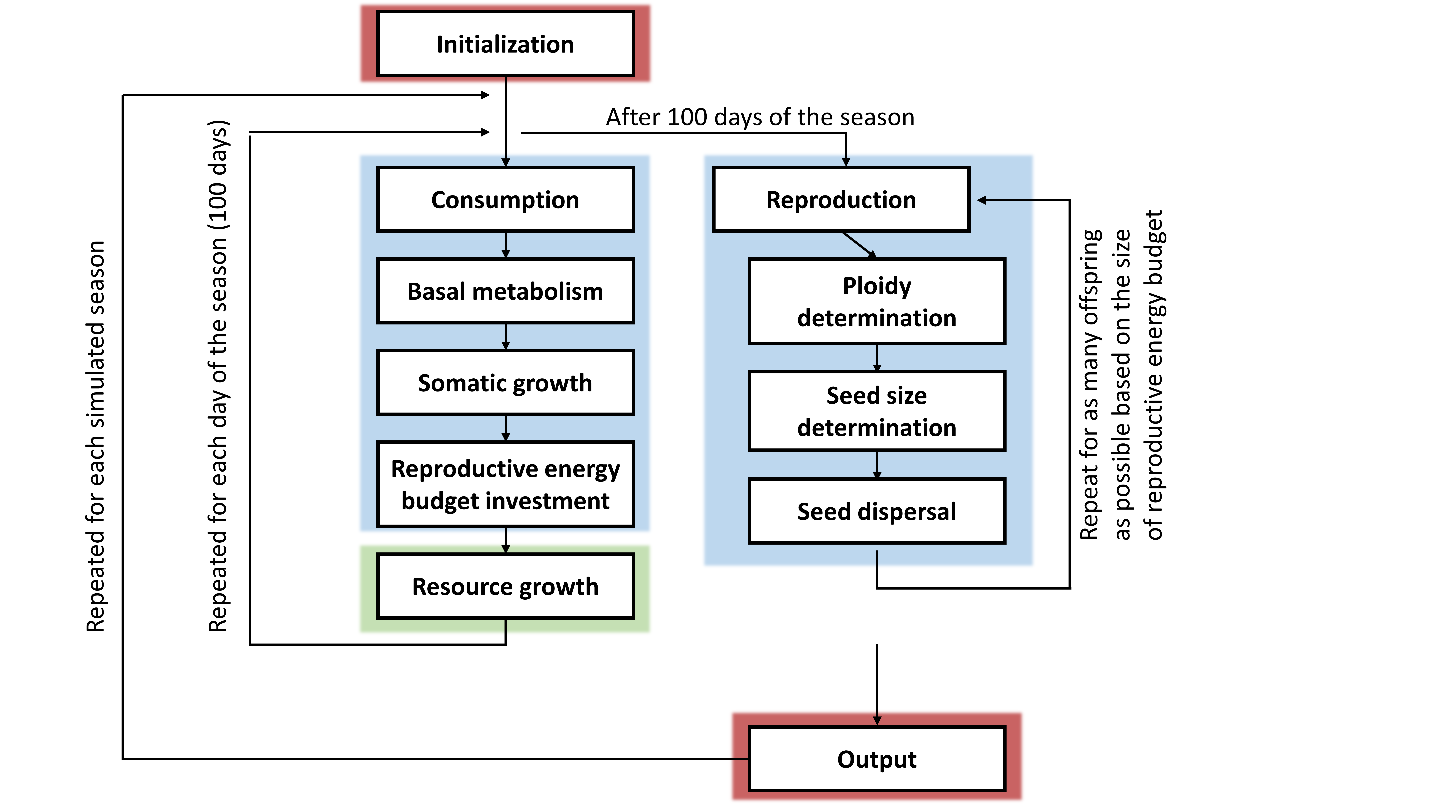


Figure S1. Depiction of all events within the model. Initialization and output generation are regulated at the level of the model (red boxes). Blue boxes represent events of the consumers and the green box represents the nutrients growth.

## ***Process overview and scheduling —***

The utilized model is a spatially explicit, discrete-time framework featuring daily growth events and seasonal reproduction events (Fig. S1). Each time step corresponds to a single day in the consumer's lifecycle, and a season is represented by a span of 100 days. In line with a plant species-based approach, we adopt a semelparous reproduction strategy, wherein reproduction occurs once in an individual's lifetime following the conclusion of a 100-day growth season. Nutrient availability is conceptualized as nitrogen- and phosphorus-limited carbon uptake for photosynthesis.

Individuals reproduce asexually, driven exclusively by their reproductive energy budget size that has accumulated throughout the season.

During each day, every individual attempts to consume nutrients from the landscape. The amount of nutrients that an individual tries to consume is related to its current mass, based on Hu et al. 2021. Their model incorporates a relationship between photosynthetic rate and carbon uptake with plant mass, accounting for nitrogen and phosphorus as limiting factors for plant growth. In instances of nutrient scarcity, individuals undergo a reduction in mass as they allocate energy to basal metabolic maintenance, essential for daily survival. Consequently, energy allocated to growth leads to reversible increases in body size. Consequently, individuals may perish due to "starvation."

When an adequate nutrient supply is available, an individual's sequence of actions involves deducting the basal metabolic maintenance "fee", subsequently allocating nutrients to somatic growth, and ultimately contributing to the reproductive energy budget. At the close of each day, nutrient levels are restored.

At the end of the season (i.e. after 100 days), individuals reproduce if their reproductive energy budget is not depleted, following which they perish. Reproducing individual allocates the REB to producing seeds of certain seed sizes (sampled from normal distribution with mean 0.5 and standard deviation 0.05), continuing this allocation until the budget is exhausted. Diploid individuals have the potential to produce tetraploid offspring, with the probability of this occurrence being sampled from a Beta distribution with a mean of 4.76%, shape parameters *α* = 2 and *β* = 40, based on previous findings (Ramsey 2007; Kreiner et al. 2017). We chose this specific distribution to reflect the observations that the distribution of naturally forming unreduced gametes rate is heavily zero-inflated and that asexual species have a higher mean than sexual or mixed-mating systems (Ramsey 2007; Kreiner et al. 2017). For simplicity, when tetraploid individuals reproduce, we assume they can only produce tetraploid offspring.

The sequence in which individuals consume does not affect the outcome, as their order is randomized before the execution of the daily events. An individual performs all the daily actions before the next individuals, and the order is unbiased by randomization of the individuals’ order at each time step (day). As the nutrients are limited (see section Landscape), individuals at the same site experience nutrient competition to acquire these nutrients. After a season of 100 days, each individual has a chance to reproduce (Fig. S1).

## ***Design concepts —***

### *Basic principles:*

One of the most commonly observed phenotypic consequences of polyploidization is an increase in cell size and overall body size (Stebbins 1971, Bomblies 2020). Body size stands as a crucial attribute in the ecophysiology of organisms. The Metabolic Theory of Ecology established the correlation between body size and numerous functional traits (Peters, 1983; Brown et al. 2004, Reich et al. 2006, Price et al. 2010), such as basal metabolic rate, ingestion rate, and developmental time. A fundamental tenet in biology is that metabolic rates scale allometrically to body size, often approximated by a scaling exponent of 0.75 (Kleiber 1932). Notably, in plant and phytoplankton taxa, respiratory rate scaling appears to deviate from the animal pattern, exhibiting a higher exponent approaching 1 (isometry, Reich et al. 2006; Lopez-Sandoval et al. 2014), even though other indicators of metabolism, such as biomass production rate, adhere to a body size exponent of 0.75 (Niklas and Enquist 2001). Given this variability, for our model, we opted to employ a distinct metabolic framework proposed by Hu et al. (2021). This model captures the relationship between photosynthetic rate and body size while also accounting for nutrient considerations. The model's fit yielded superior explanatory power for plant data compared to the allometric scaling model, prompting our decision to adopt it. We employ their estimation for non-woody plants as a formula governing the daily amount of photosynthesis (consumption) in a plant.

Due to having twice the amount of DNA as their diploid predecessors, tetraploids are postulated to necessitate increased energy and nutrient allocation for basal metabolic maintenance. However, this heightened allocation could potentially limit nutrients available for somatic and reproductive growth. As a reference, we regard diploid energy and nutrient utilization as the most metabolically efficient, characterized by a distribution of 36% for basal metabolic maintenance, 54% for somatic growth, and 10% for the reproductive energy budget (36:54:10). Consequently, we investigate the implications of varying metabolic efficiencies in tetraploids on population dynamics and the establishment of tetraploids. This is achieved by altering the 36:54:10 ratio to reflect lower metabolic efficiencies in tetraploids—for instance, 37:54:9, indicating a 10% reduction in efficiency compared to diploids.

The notion that polyploids may exhibit suppressed metabolism was postulated by Cavalier-Smith (1978), who drew from data obtained from *Ribes satigrum*, *Datura stramonium*, *Raphanus sativus*, *Galeopsis pubescens*, *Solanum nudiflorum*, *Vitis vinifera*, and *Phlox drummondii*. In these cases, CO_2_ exchange rates decreased with increases in ploidy levels, possibly due to alterations in the geometry of polyploid cells. We explore the implications of these differences in metabolic efficiency across three distinct life history strategies: annual individuals, perennial individuals, and annual diploids coexisting with perennial tetraploids. The outcomes of these diverse models are then subjected to comparison.

While it is generally anticipated that polyploids would exhibit diminished metabolic rates in contrast to diploids, there are instances that deviate from this trend (Levin 1983), indicating potential increases in metabolic rates among polyploids. We consequently simulated these variations in metabolic efficiency as well.

### *Emergence:*

Population dynamics emerge from the behavior of the individuals, where the daily life cycle is represented by empirical rules describing metabolism. Successfully reproducing diploids can have diploid and tetraploid offspring, as there is a probability (Beta distribution with mean 4.76%, shape parameters *α* = 2 and *β* = 40) for each offspring to be tetraploid. With no inherent differentiation between diploids and tetraploids, the tetraploid accumulation is anticipated due to their role as a sink.

Nutrient distribution undergoes spatial alterations as individuals consume nutrients daily, amplifying the significance of competition. Smaller individuals require fewer nutrients, which may allow multiple individuals in a cell to sustain growth and investment in the reproductive energy budget. Conversely, larger individuals exhibit heightened resistance to starvation, as their greater mass provides a buffer against nutrient scarcity. Disparities in metabolic efficiency between diploids and tetraploids are poised to shape population dynamics, contributing to the emergence of distinct body sizes.

Furthermore, variations in life history strategies are anticipated to yield diverse population dynamics. In the context of annuals, which experience only a single season, those individuals that secure optimal nutrients and allocate them to the reproductive energy budget attain the highest absolute fitness. In contrast, the perennial strategy boasts advantages as reproduction is postponed to subsequent seasons, permitting individuals to accumulate more nutrients and potentially augment their reproductive energy budget.

As a result, the distribution of body sizes will materialize as an outcome of nutrient availability and metabolic efficiencies, intertwined with life strategies. Together, these factors culminate in determining population dynamics, including the potential coexistence of tetraploids with diploids or the invasion of tetraploids.

### *Adaptation:*

In this model, body size and ploidy stand as the principal defining characteristics of individuals. The rate of consumption depends on body size, resulting in dynamic changes attributable to the allocation among basal metabolic maintenance, somatic growth, and the reproductive energy budget.

Within the confines of a shared cell, individuals engage in competition for limited resources. This competition yields instances where some individuals consume less than the optimal amount of nutrients, leading to a loss in their reproductive energy budget and somatic mass. It's important to note that adaptation is not a feature of this model, as it lacks an inheritance mechanism.

### *Stochasticity:*

All demographic parameters are treated as probabilities or are drawn from probability distributions. This approach was adopted to include demographic noise and emphasize population-level phenomena, rather than individual behavior.

### *Observation:*

During each time step, we recorded the counts of individuals (both diploid and tetraploid) and nutrient levels across all cells, alongside the masses of individuals. Upon reproduction, the ages and masses of reproducing individuals were also documented. The spatial distribution of individuals was visualized to provide a comprehensive perspective.

## ***Details —***

### *Initialization:*

For each parameter combination, 10 simulations were run, each with 10000 time steps or 100 seasons. At the start of each simulation, 1600 diploids were introduced as seeds and randomly distributed across the grid cells of the habitat. Every grid cell was initiated with 1 gram of nutrients. The initial number of individuals was selected to maintain an average density is one individual per cell.

### Input data:

Seed sizes for each individual are drawn from a normal distribution with a mean of 0.5 g and a standard deviation of 0.05 g. Background mortality was kept in all submodels, with a rate of 5 individuals per 1000.

### *Submodels:*

Case 1: all individuals are annuals so whether or not they have filled their reproductive energy budget after 100 days, they die.

Case 2: all individuals are perennials, which means that they are allowed to live through multiple seasons until they can produce offspring unless they die in the meantime.

Case 3: polyploids are perennial and diploids are annual, meaning polyploids can live for multiple seasons and have multiple opportunities for reproduction, whereas diploids die after a single growing season.

In total, simulations are done for metabolic efficiency where polyploids can be as efficient as diploids or less or more, for each of the three cases of annual/perennial reproductive strategies.

Table S1. Set of parameters used in the model simulations developed in this study. The table contains the parameter name and symbol, the parameter values used, and a brief description of the biological interpretation.

| Parameter | Value and unit | Biological interpretation |
| --- | --- | --- |
| *Ploidy* | 2 or 4 | Diploid or tetraploid individuals |
| *Parental ploidy* | 2 or 4 | Tetraploid individuals may have diploid or tetraploid parents |
| *Seed mass W0* | Sampled from Normal distribution Normal(0.5, 0.05), in grams | Each seed is formed with the initial mass sampled from this normal distribution |
| *Somatic mass W* | In grams | Each individual grows in its somatic mass starting from the seed mass and depending on the photosynthetic rate and nutrient uptake |
| *Reproductive energy reserve* | In grams | Initially zero for each offspring, and during simulations increases and decreases depending on the photosynthetic rate and nutrient availability |
| *Mass M* | In grams | Total mass of an individual, including somatic mass, and reproductive energy reserve |
| *Season* | 100 days | Reproduction happens at the end of a season only, whereas nutrient uptake, photosynthesis and metabolism happen on all days |
| *Polyploid formation* | Sampled from Beta distribution B(1, 20) with mean of 0.0476 | Each offspring has this probability of becoming a polyploid |
| *x, y* | 0-39 | As the simulated space is 40x40 cells, the position of an individual is (*x*,*y*) where both x and y are within the range of 40 numbers |
| *Nutrients* | In grams | Initially 10 g of nutrients in each simulated cell, and then increase and decrease based on the individuals consuming from the cells |
| *Nutrients growth rate* | *r* = 1 g/day |  |
| *Maximum nutrients content* | *Rmax* = 10 g per cell |  |
| *Initial number of individuals* | 1600 diploids |  |
| *Time to reproduction* | Number of seasons |  |
| *Mass at reproduction* | In grams |  |
| *Background mortality* | 0.0005 | Random background mortality per day, meaning 5 in 10000 individuals die due to this chance event |
| *Photosynthetic rate Q* | *Q* = (*QM* ×*M*)/(*kQM*+ *θ* × *M*)  *Q* = 32.8 * *M* / (1.06 + 0.94 * *M*)  Units µmol/s | Based on Hu et al. (2021) model and their results for non-woody plants, where *M* is the current mass of the individual |
| *Basal metabolism BMR* | Diploids: 0.36 *Q*  Tetraploids: 0.36 *Q*, 0.361 *Q*, …, 0.369 *Q*, 0.37 *Q* | Explanations for these choices are given in the text |
| *Somatic growth* | 0.54 *Q* |  |
| *Reproductive energy budget* | Diploids: 0.10 *Q*  Tetraploids: 0.10 *Q*, …, 0.09 *Q* |  |

## Literature Cited

Grimm, V., Berger, U., Bastiansen, F., Eliassen, S., Ginot, V., Giske, J., Goss-Custard, J. et al. (2006). A standard protocol for describing individual-based and agent-based models. *Ecological* *Modelling*, 198(1), 115–126. <https://doi.org/https://doi.org/10.1016/j.ecolmodel.2006.04.023>

Grimm, V., Berger, U., DeAngelis, D. L., Polhill, J. G., Giske, J., and Railsback, S. F. (2010). The ODD protocol: A review and first update. *Ecological Modelling*, 221(23), 2760–2768. <https://doi.org/https://doi.org/10.1016/j.ecolmodel.2010.08.019>

Grimm, V., Railsback, S. F., Vincenot, C. E., Berger, U., Gallagher, C., DeAngelis, D. L., Edmonds, B. et al. (2020). The ODD Protocol for Describing Agent-Based and Other Simulation Models: A Second Update to Improve Clarity, Replication, and Structural Realism*. Journal of Artificial Societies and Social Simulation*, 23(2), 7. <https://doi.org/10.18564/jasss.4259>

Hu, H.-J., Xu, K., He, L.-C., and Wang, G.-X. (2021). A model for the relationship between plant biomass and photosynthetic rate based on nutrient effects. *Ecosphere*, 12(8), e03678. <https://doi.org/https://doi.org/10.1002/ecs2.3678>

Ramsey, J. (2007). Unreduced gametes and neopolyploids in natural populations of Achillea borealis (Asteraceae). *Heredity*, 98(3), 143–150. <https://doi.org/10.1038/sj.hdy.6800912>

Kreiner, J. M., Kron, P., and Husband, B. C. (2017a). Evolutionary Dynamics of Unreduced Gametes. *Trends in Genetics*, 33(9), 583–593. <https://doi.org/https://doi.org/10.1016/j.tig.2017.06.009>

Stebbins, G.L. (1971) Chromosomal Evolution in Higher Plants. Edward Arnold LTD, London, UK

Bomblies, K. (2020). When everything changes at once: finding a new normal after genome duplication. *Proceedings of the Royal Society, B, Biological Sciences*, 287(1939), 20202154. <https://doi.org/10.1098/rspb.2020.2154>

Peters, R. H. (1983). The Ecological Implications of Body Size. Cambridge Studies in Ecology. Cambridge: Cambridge University Press. <https://doi.org/DOI:10.1017/CBO9780511608551>

Price, C. A., Gilooly, J. F., Allen, A. P., Weitz, J. S., and Niklas, K. J. (2010). The metabolic theory of ecology: prospects and challenges for plant biology. *New Phytologist*, 188(3), 696–710. <https://doi.org/https://doi.org/10.1111/j.1469-8137.2010.03442.x>

Brown, J. H., Gillooly, J. F., Allen, A. P., Savage, V. M., and West, G. B. (2004). Toward A Metabolic Theory of Ecology. *Ecology*, 85(7), 1771–1789. <https://doi.org/https://doi.org/10.1890/03-9000>

Reich, P. B., Tjoelker, M. G., Machado, J.-L., and Oleksyn, J. (2006). Universal scaling of respiratory metabolism, size and nitrogen in plants. *Nature*, 439(7075), 457–461. <https://doi.org/10.1038/nature04282>

Kleiber, M. (1932). Body size and metabolism. *Hilgardia* 6, 315–353. <https://doi.org/10.3733/hilg.v06n11p315>

Lopez-Sandoval, D. C., Rodríguez-Ramos, T., Cermeño, P., Sobrino, C., and Marañón, E. (2014). Photosynthesis and respiration in marine phytoplankton: Relationship with cell size, taxonomic affiliation, and growth phase. *Journal of Experimental Marine Biology and Ecology*, 457, 151–159. <https://doi.org/https://doi.org/10.1016/j.jembe.2014.04.013>

Niklas, K. J., and Enquist, B. J. (2001). Invariant scaling relationships for interspecific plant biomass production rates and body size. *Proceedings of the National Academy of Sciences*, 98(5), 2922–2927. <https://doi.org/10.1073/pnas.041590298>

Cavalier-Smith, T. (1978). Nuclear volume control by nucleoskeletal DNA, selection for cell volume and cell growth rate, and the solution of the DNA C-value paradox. *Journal of Cell Science*, 34(1), 247–278. <https://doi.org/10.1242/jcs.34.1.247>

Levin, D. A. (1983). Polyploidy and Novelty in Flowering Plants. *The American Naturalist*, 122(1), 1–25. <https://doi.org/10.1086/284115>
