# Supplementary material for: A metabolic perspective on polyploid invasion and the emergence of life histories: insights from a mechanistic model
Source: Am J Bot. Author manuscript; Available in PMC 2025 Feb 1. (PMC7616395; doi:10.1002/ajb2.16387)
Supplement: Appendix 2 [file EMS198114-supplement-Appendix_2.docx]

# Appendix S2

These figures are supplementary results of the model analysis. First, the dynamics without introduced polyploids is explored (Fig. S2), and then the results for spatial distribution (Fig. S3) and polyploid seed parentage (Fig. S4) are presented. Figures S5 and S6 are part of the sensitivity analysis, where we tested the results of the simulations with different polyploid formation probability distributions. Importance of recurrent polyploid formation was explored and results are presented in Fig. S7. Finally, the age distributions of diploids and tetraploids are presented in Fig. S8, where total population (A) and reproducing individuals (B) are presented separately.


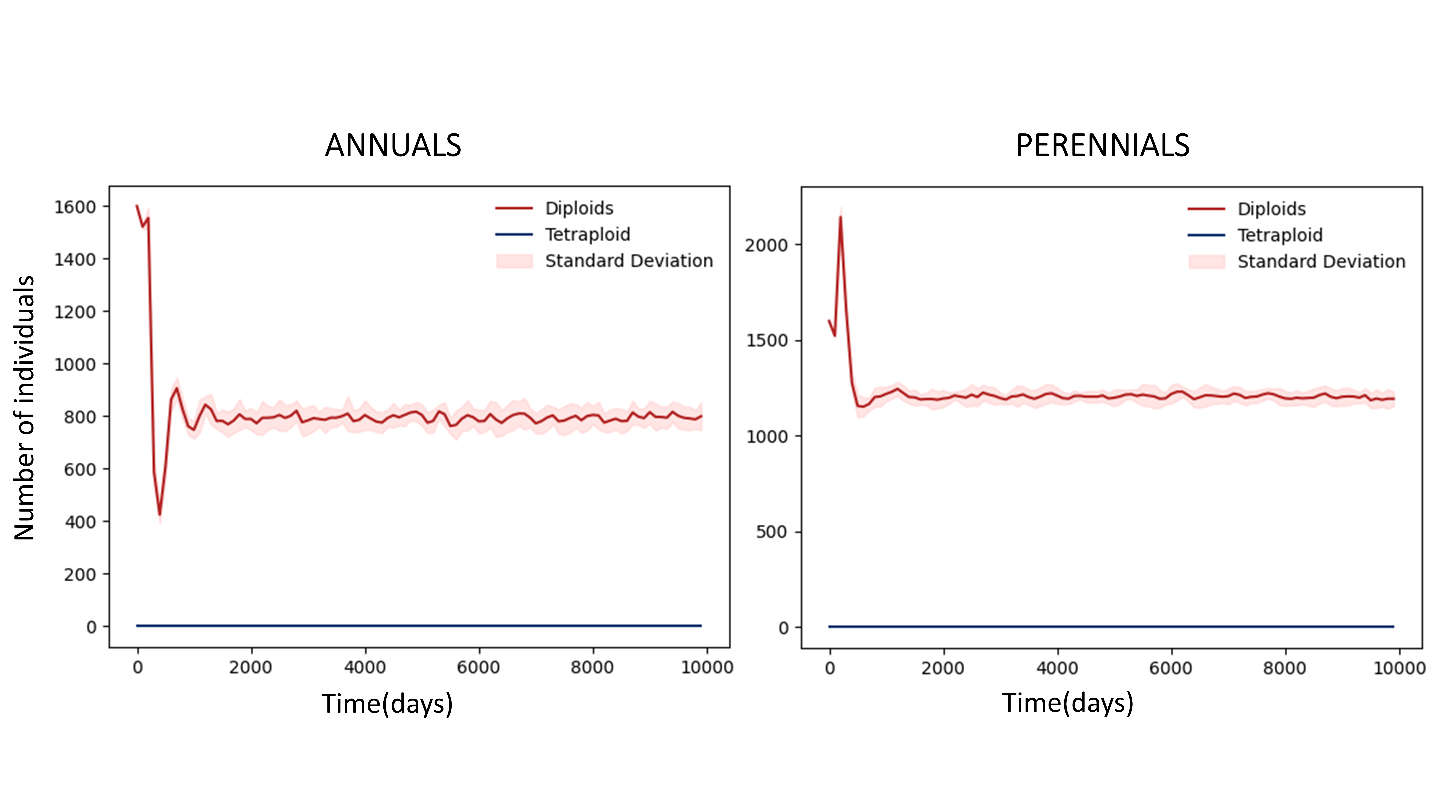


Figure S2. The number of individuals through 10000 days, without polyploid formation allowed in the simulations. The color of lines is differentiating between diploids (depicted in red) and tetraploids (depicted in blue). Results are shown for the two opposing strategies, i.e. annuality and perenniality. Notice that these plots illustrate the mean of independent simulation in darker color shades, while the lighter shade is the standard deviation. The estimated carrying capacity after stabilization was 814 and 1237 for annuals and perennials, respectively.


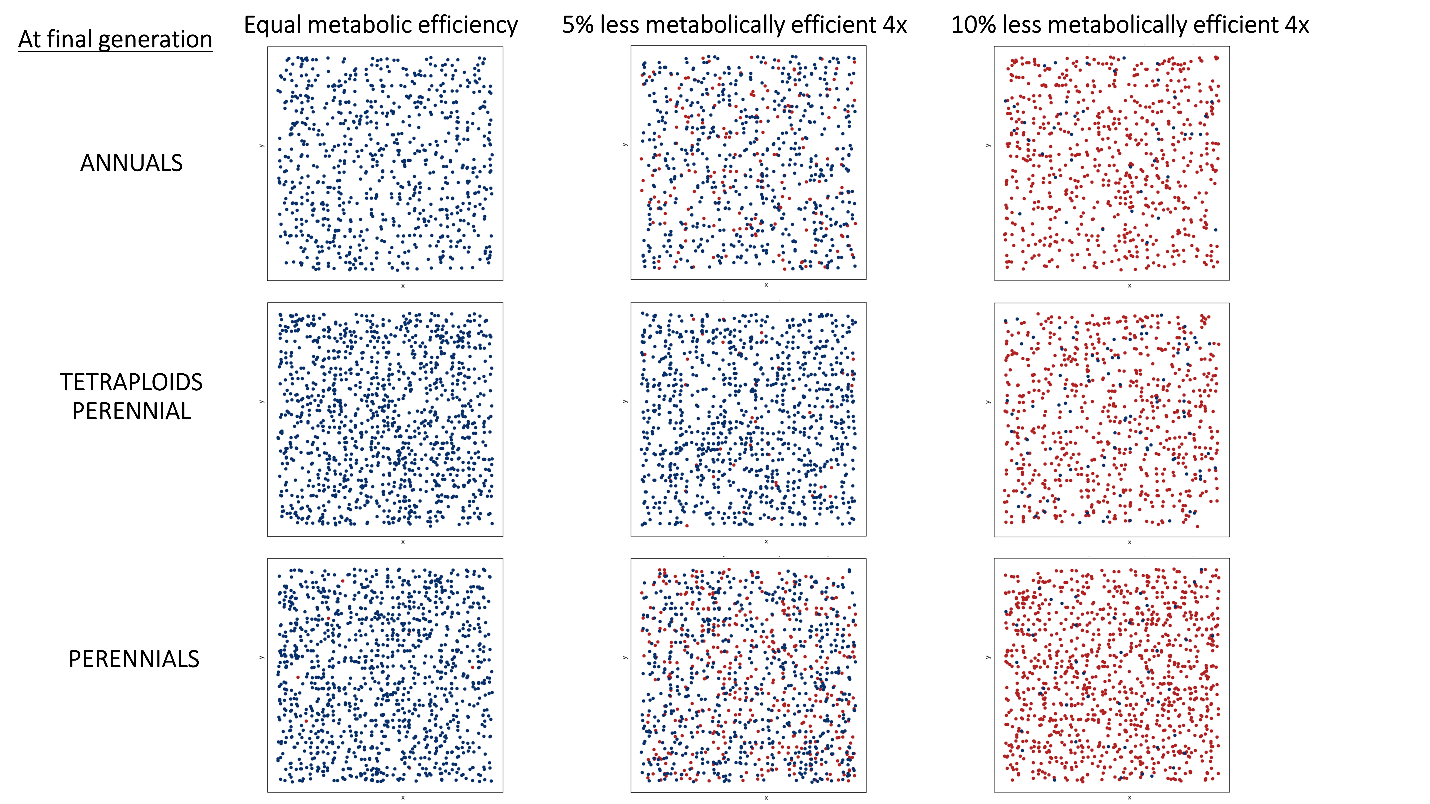


Figure S3. Spatial distribution of individuals across simulated scenarios and metabolic efficiencies. Individuals are represented as dots in (*x*, *y*) space, with the color of dots differentiating between diploids (depicted as red dots) and tetraploids (depicted as blue dots). Different rows correspond to the three life history scenarios, with the top row corresponding to the annuals scenario, the bottom row to the perennials scenario, and the middle to the scenario where only tetraploids exhibit perenniality. Different columns represent tested metabolic efficiencies, with the first column showing results when tetraploids have an equal metabolic efficiency as diploids, and the second and third columns showing results when tetraploids are 5 and 10% less efficient than diploids, respectively. Notice that these plots illustrate the results from a single run of the simulation, at the final generation (generation 100).


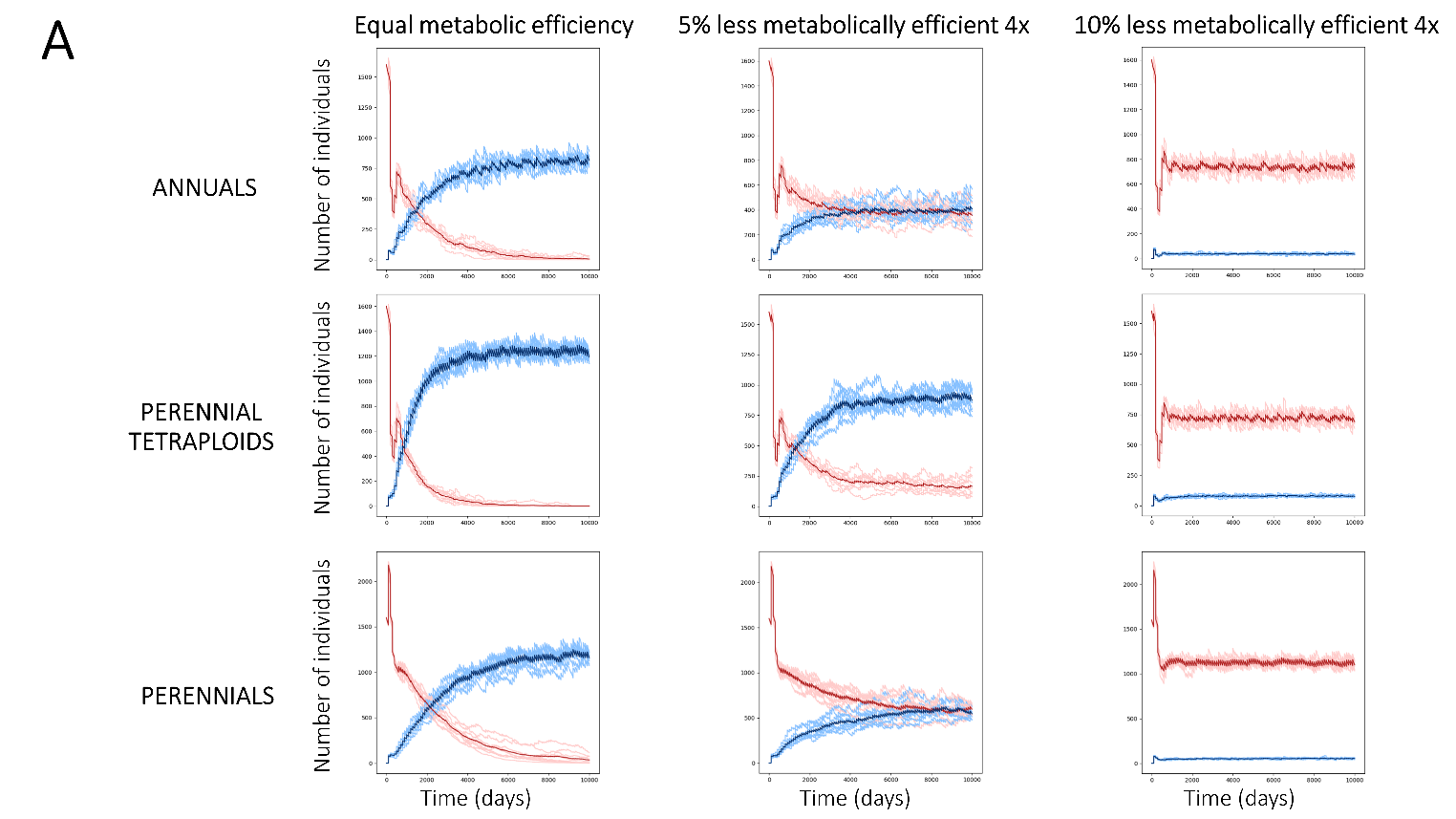


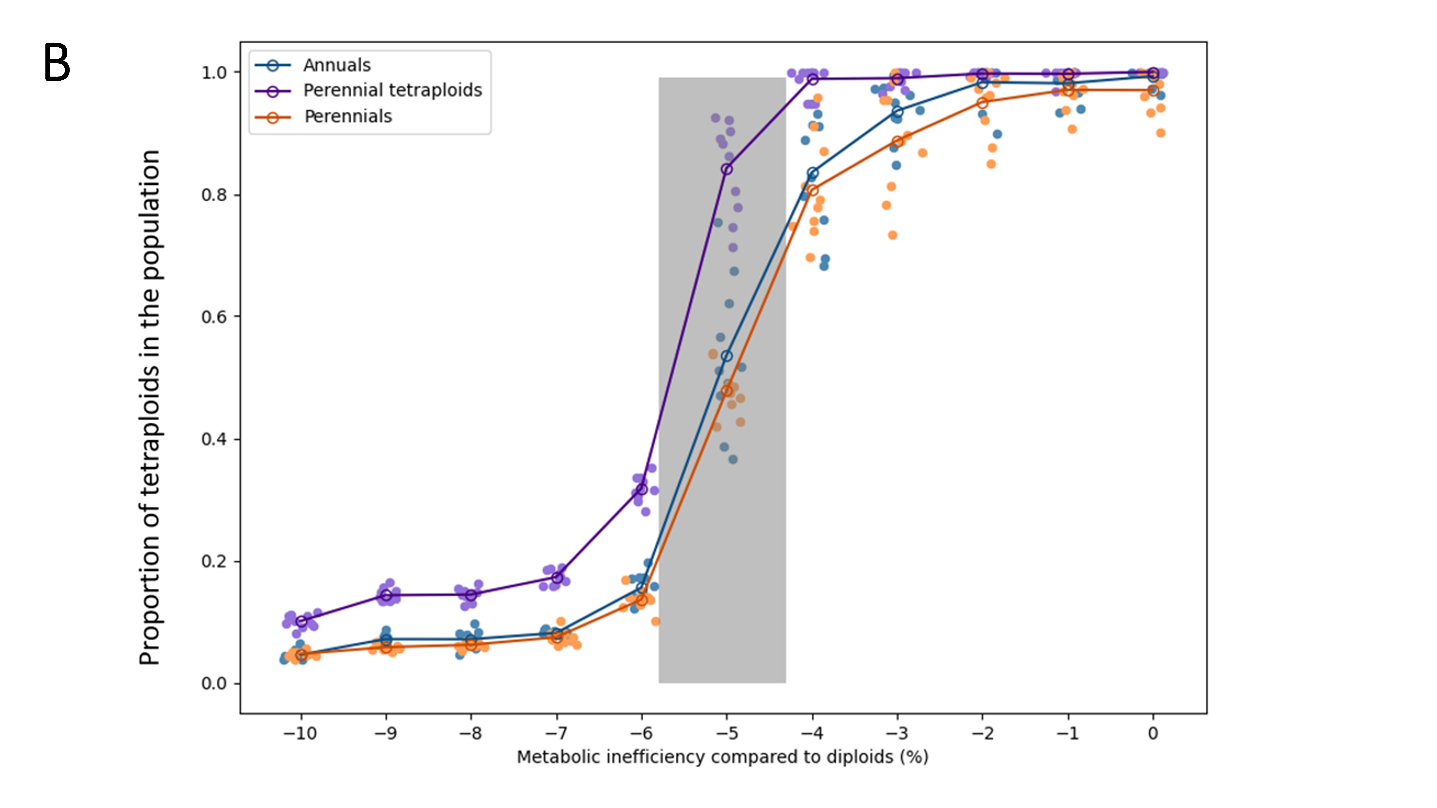


Figure S4. Main results of model simulations when polyploid formation is not drawn from Beta distribution with mean of 4.76% Beta(1, 20) but with a constant of 4.76%. A) Shows the number of individuals through 10000 days, across simulated scenarios and metabolic efficiencies. The colors are red for diploids and blue for tetraploids. Rows correspond to the three life history scenarios: top row to the annuals scenario, bottom row to the perennials scenario, and middle to the scenario where only tetraploids exhibit perenniality. Columns represent tested metabolic efficiencies, with the first column showing results when tetraploids have an equal metabolic efficiency as diploids, and the second and third columns showing results when tetraploids are 5 and 10% less efficient than diploids, respectively. Notice that these plots illustrate the mean of independent simulation in darker color shades of red and blue with applied jitter along the x-axis for visibility, while the lighter shades are all the independent simulations. B) Proportion of tetraploid individuals across metabolic inefficiencies of tetraploids in comparison to diploids. Results are shown for the three simulated scenarios: all individuals annual in blue, all individuals perennial in orange, and tetraploid perennials in purple. Independent simulation runs are represented by scattered dots, and darker shades are the means connected with lines. Notice the grey shaded area on the plot which represents the zone where coexistence is observed.


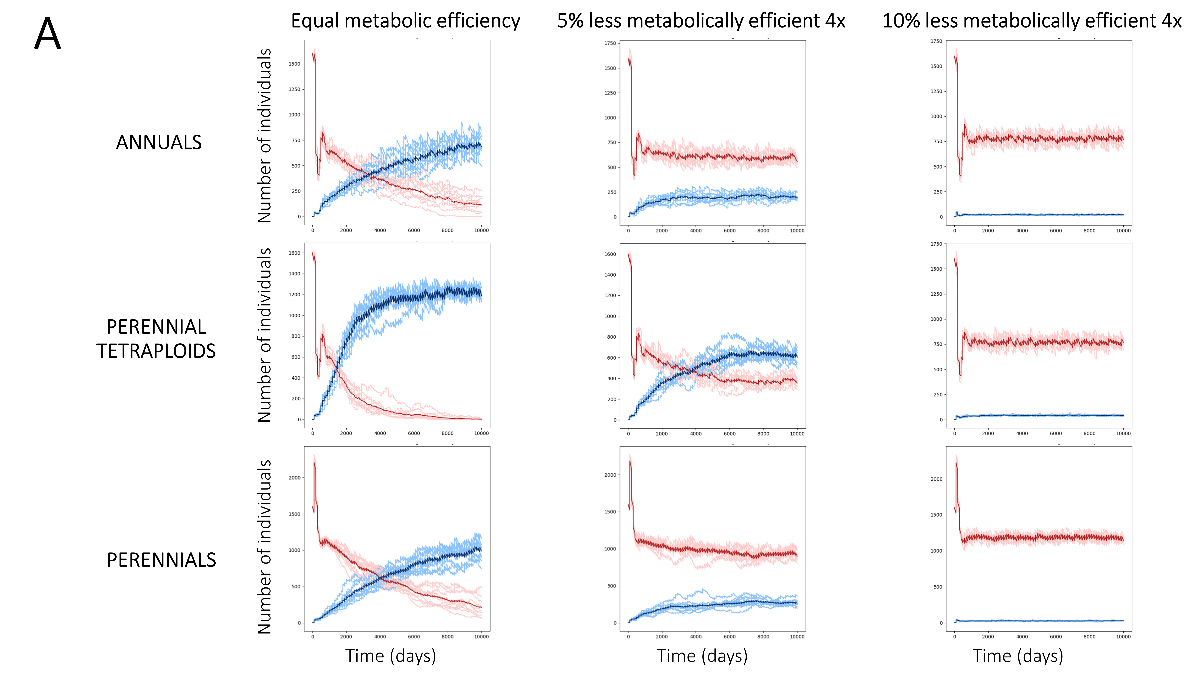


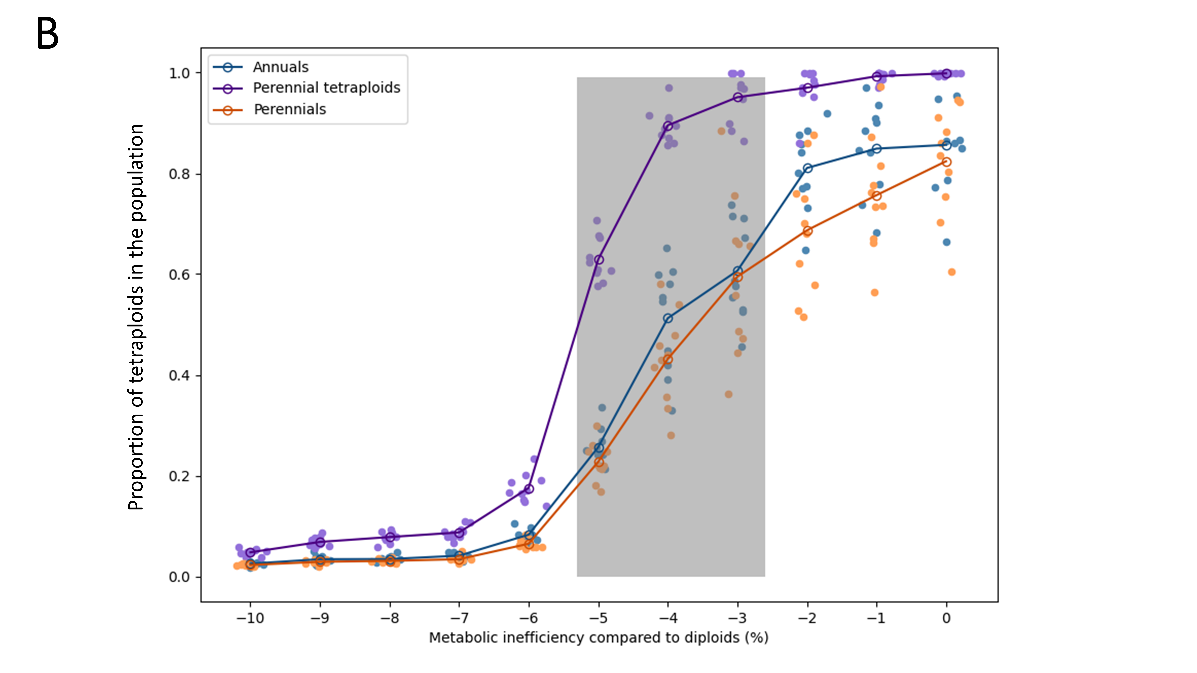


Figure S5. Main results of model simulations when polyploid formation is drawn from Beta distribution with mean of 2.38% Beta(2, 82). A) Shows the number of individuals through 10000 days, across simulated scenarios and metabolic efficiencies. The colors are red for diploids and blue for tetraploids. Rows correspond to the three life history scenarios: top row to the annuals scenario, bottom row to the perennials scenario, and middle to the scenario where only tetraploids exhibit perenniality. Columns represent tested metabolic efficiencies, with the first column showing results when tetraploids have an equal metabolic efficiency as diploids, and the second and third columns showing results when tetraploids are 5 and 10% less efficient than diploids, respectively. Notice that these plots illustrate the mean of independent simulation in darker color shades of red and blue, while the lighter shades are all the independent simulations. B) Proportion of tetraploid individuals across metabolic inefficiencies of tetraploids in comparison to diploids. Results are shown for the three simulated scenarios: all individuals annual in blue, all individuals perennial in orange, and tetraploid perennials in purple. Independent simulation runs are represented by full dots with applied jitter along the x-axis for visibility, and darker open dots are the means connected with lines. Notice the grey shaded area on the plot which represents the zone where coexistence is observed.


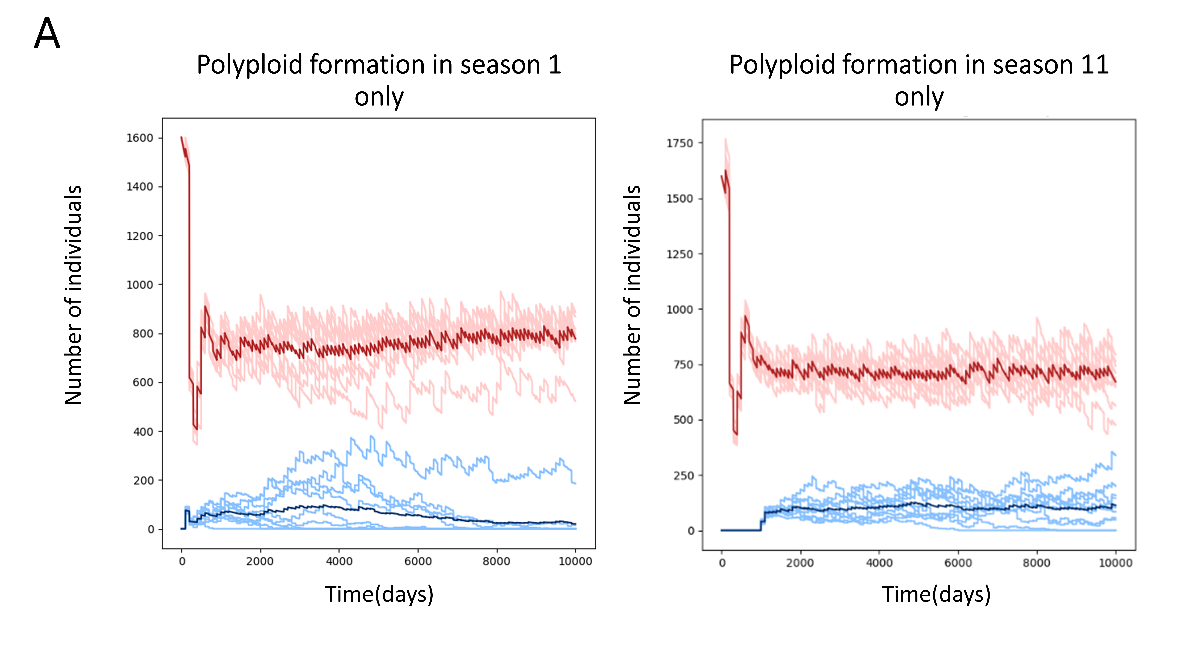


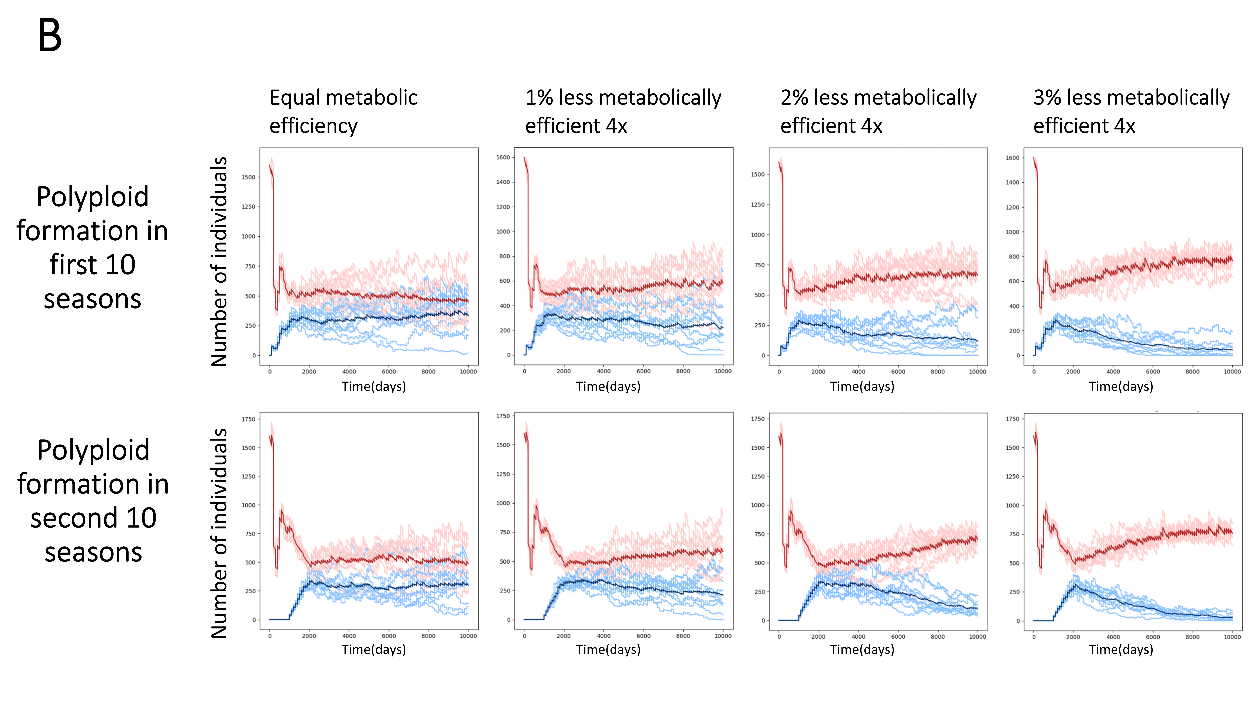


Figure S6. Number of individuals through 10000 days, across scenarios where all individuals are annuals and polyploid formation is not recurrent or constant. The color of lines is differentiating between diploids (depicted in red) and tetraploids (depicted in blue). Notice that these plots illustrate the mean of independent simulation in darker color shades of red and blue, while the lighter shades are all the independent simulations. A) shows the number of individuals when polyploids are equally metabolically efficient as diploids, but polyploid formation is allowed only during one season, either the first simulated season (left) or the eleventh season when the diploid population reaches carrying capacity (right). B) shows the number of individuals when polyploid formation is allowed only during 10 seasons, either the first 10 seasons (top) or the second 10 seasons when the diploid population reached carrying capacity (bottom). The different columns represent tested metabolic efficiencies, with the first column showing results when tetraploids have an equal metabolic efficiency as diploids, and towards the right are lower efficiencies of polyploids one by one percent less than in diploids.


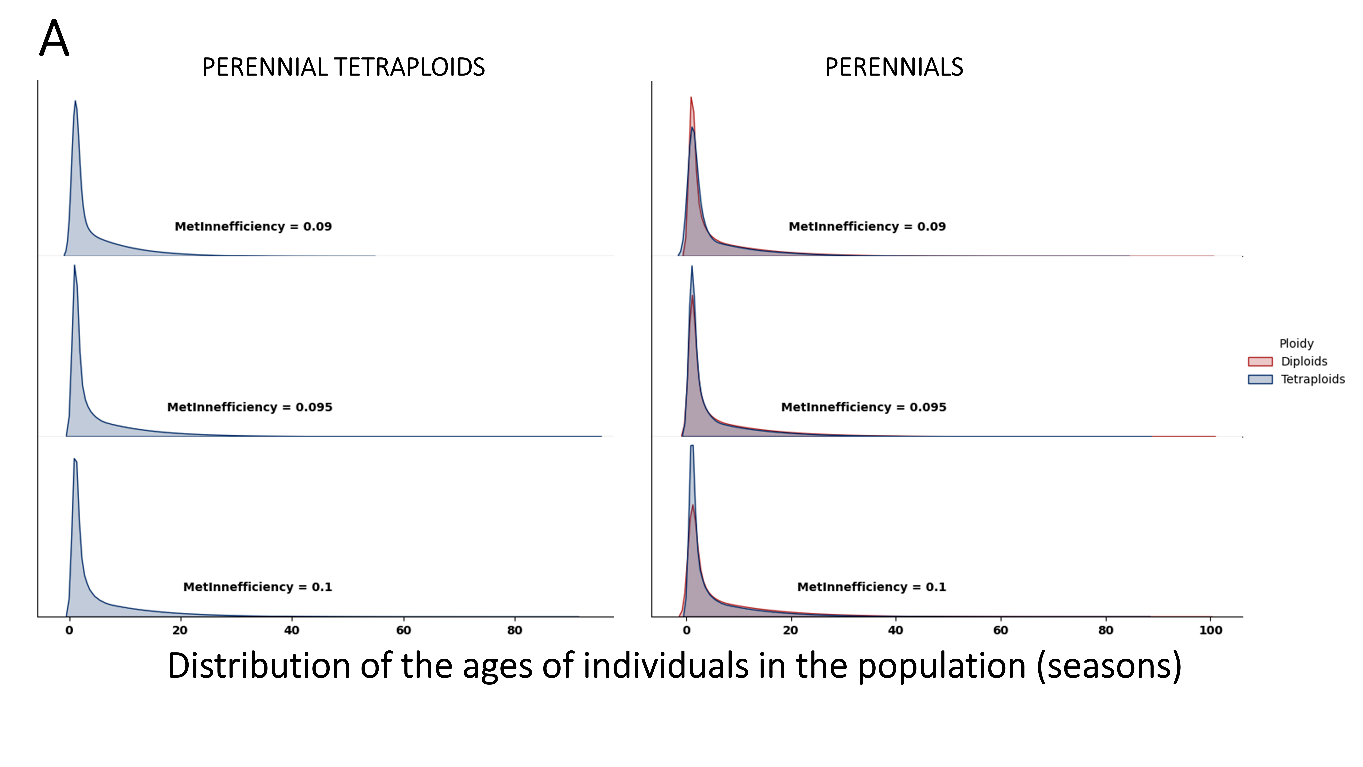


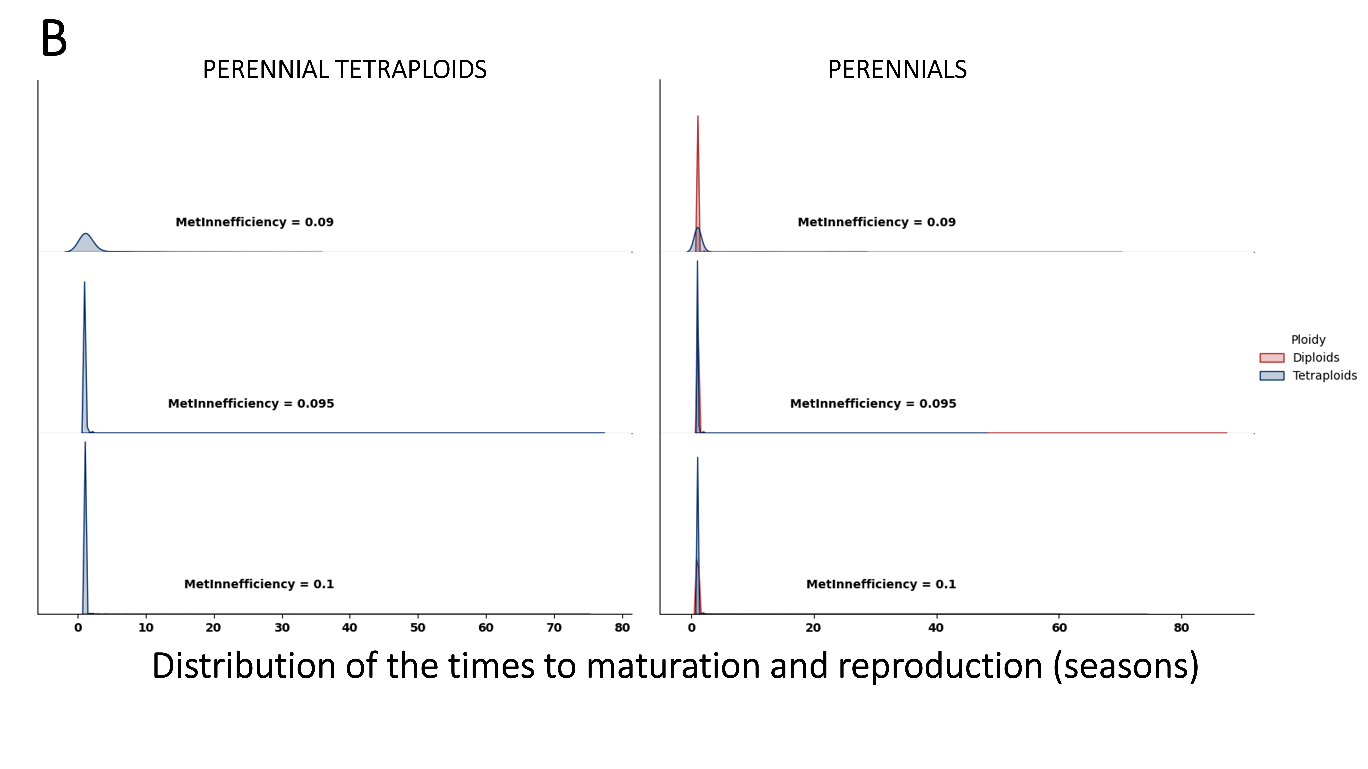


Figure S7. Distribution of ages of diploid and tetraploid individuals across simulated metabolic inefficiencies of tetraploids and life history strategies. A) Shows the age distributions of all individuals, whereas B) shows the age distributions of only the individuals which matured and successfully reproduced. Columns correspond to the tested life history scenarios. Rows correspond to the tested metabolic inefficiencies of tetraploids in comparison to diploids, with the top and middle rows where tetraploids are 10% and 5% less efficient, respectively, and the bottom row where they are equal.
